# Optimization and Validation of a Novel Three-Dimensional Co-Culture System in Decellularized Human Liver Scaffold for the Study of Liver Fibrosis and Cancer

**DOI:** 10.3390/cancers13194936

**Published:** 2021-09-30

**Authors:** Kessarin Thanapirom, Elisabetta Caon, Margarita Papatheodoridi, Luca Frenguelli, Walid Al-Akkad, Zhang Zhenzhen, Maria Giovanna Vilia, Massimo Pinzani, Giuseppe Mazza, Krista Rombouts

**Affiliations:** 1 Regenerative Medicine and Fibrosis Group, Institute for Liver and Digestive Health, University College London, Royal Free Campus, London NW3 2PF, UK; kessarin.t@chula.ac.th (K.T.); e.caon@ucl.ac.uk (E.C.); m.papatheodoridi@ucl.ac.uk (M.P.); l.frenguelli@alumni.ucl.ac.uk (L.F.); Walid.al-akkad@ucl.ac.uk (W.A.-A.); zhangzhenzhen@cqmu.edu.cn (Z.Z.); m.vilia@ucl.ac.uk (M.G.V.); m.pinzani@ucl.ac.uk (M.P.); Giuseppe.mazza.12@ucl.ac.uk (G.M.); 2 Division of Gastroenterology, Department of Medicine, Liver Fibrosis and Cirrhosis Research Unit, Faculty of Medicine, Chulalongkorn University and King Chulalongkorn Memorial Hospital, Thai Red Cross Society, Bangkok 10330, Thailand; 3 Sheila Sherlock Liver Centre, Royal Free Hospital, London NW3 2QG, UK

**Keywords:** decellularized liver scaffolds, drug screening, liver fibrosis, liver cancer, sorafenib, regorafenib, STAT3, SHP-1, TGFβ1, EMT, E-cadherin, 3D in vitro model

## Abstract

**Simple Summary:**

This study aims to overcome the current methodological limitations in discovering new therapeutic targets. Therefore, we optimized and validated a co-culture system using decellularized human liver three-dimensional (3D) scaffolds obtained from healthy and cirrhotic human livers for anti-fibrotic and anti-cancer dual drug screening. Both platforms mimic the naturally healthy and physio-pathological microenvironment and are able to recapitulate the key cellular and molecular events leading to liver fibrogenesis and cancer. This study demonstrates the differences between single versus co-cultures and the usage of human-derived liver 3D ECM scaffolds from healthy and cirrhotic livers. As lead compounds, we used Sorafenib and Regorafenib, first- and second-line drugs, and identified two different drug-induced mechanisms depending on the 3D ECM microenvironment. The 3D ECM scaffolds may represent innovative platforms for disease modeling, biomarker discovery, and drug testing in fibrosis and primary cancer.

**Abstract:**

The introduction of new preclinical models for in vitro drug discovery and testing based on 3D tissue-specific extracellular matrix (ECM) is very much awaited. This study was aimed at developing and validating a co-culture model using decellularized human liver 3D ECM scaffolds as a platform for anti-fibrotic and anti-cancer drug testing. Decellularized 3D scaffolds obtained from healthy and cirrhotic human livers were bioengineered with LX2 and HEPG2 as single and co-cultures for up to 13 days and validated as a new drug-testing platform. Pro-fibrogenic markers and cancer phenotypic gene/protein expression and secretion were differently affected when single and co-cultures were exposed to TGF-β1 with specific ECM-dependent effects. The anti-fibrotic efficacy of Sorafenib significantly reduced TGF-β1-induced pro-fibrogenic effects, which coincided with a downregulation of STAT3 phosphorylation. The anti-cancer efficacy of Regorafenib was significantly reduced in 3D bioengineered cells when compared to 2D cultures and dose-dependently associated with cell apoptosis by cleaved PARP-1 activation and P-STAT3 inhibition. Regorafenib reversed TGF-β1-induced P-STAT3 and SHP-1 through induction of epithelial mesenchymal marker E-cadherin and downregulation of vimentin protein expression in both co-cultures engrafting healthy and cirrhotic 3D scaffolds. In their complex, the results of the study suggest that this newly proposed 3D co-culture platform is able to reproduce the natural physio-pathological microenvironment and could be employed for anti-fibrotic and anti-HCC drug screening.

## 1. Introduction

Liver fibrosis is characterized by excessive extracellular matrix (ECM) accumulation in response to chronic liver injury, with possible progression to cirrhosis and hepatocellular carcinoma (HCC). More than 70% of HCC arise in fibrotic or cirrhotic livers, suggesting that the fibrotic transformation of liver ECM tissue represents an important pre-neoplastic condition [1,2,3,4]. Indeed, it is well established that targeting exclusively tumor cells does not significantly affect patient survival and recent studies demonstrated that the tumor microenvironment ECM and stromal cells profoundly affect tumor cell behavior [5]. In spite of a significant understanding of the mechanisms responsible for liver fibrosis and HCC, there are no approved anti-fibrotic agents and there are few anti-cancer agent strategies able to significantly reduce HCC progression [6,7]. A major obstacle in the development of new agents is the lack of appropriate in vitro models for liver fibrosis and HCC recapitulating the pathophysiological micro-environmental complexity of these clinical conditions in humans.

Currently, two-dimensional (2D) monolayer single-cell culture systems are commonly used as in vitro models for the study of liver fibrosis and cancer. However, 2D cell culture models have several limitations, including lack of complex three-dimensional (3D) architecture, cell–cell or cell–ECM interactions, and the induction of artifactual biomechanical effects by the plastic culture conditions [8,9]. For example, culturing freshly isolated quiescent hepatic stellate cells (HSCs) on a 2D plastic culture dish leads to spontaneous activation and transdifferentiation into myofibroblast-like cells due to the high surface tension (approximately 20 Giga Pa) of the plastic surface [10,11]. This is in strong contrast with the cell–tissue tension measured in healthy and cirrhotic human decellularized liver, i.e., 2 kPa and 5 kPa, respectively [12]. This is further supported by the significantly greater drug resistance observed in 3D co-culture cell models, such as spheroids or collagen gels, compared to what was previously observed in 2D models, indicating that tumor microenvironment (TME) and tissue tension can represent essential factors affecting cancer cell behavior and susceptibility to treatment [13,14].

In the past decade, the introduction of 3D culture has rapidly progressed with the development of more complex in vitro models [15,16,17]. Our group developed a novel 3D in vitro model of decellularized human liver scaffolds by using healthy human liver tissue unsuitable for transplantation [18]. Repopulation of these 3D scaffolds with human hepatic parenchymal and non-parenchymal cells was demonstrated to maintain cell- and tissue-specific phenotypes. For example, HepG2 cells, a human hepatoblastoma cell line reproducing many of the features of polarized human hepatocytes [19] and largely employed as a cellular component for bioartificial livers [20] maintained features of higher cellular differentiation features. Similarly, LX2 cells, a human hepatic stellate cell line, retained the phenotype of activated myofibroblast cells with important differences at mRNA expression of key pro-fibrogenic genes when compared with cells grown on standard 2D-plastic conditions [18]. Recently, we showed that healthy and cirrhotic liver 3D ECM scaffolds retained architectural, biochemical, and biomechanical features able to affect cell behavior and function with distinct patterns. In particular, both decellularized 3D ECM platforms differed in terms of unique ECM components with specific bioactive cues, microarchitecture, and tissue stiffness [12]. In cirrhotic 3D scaffolds, the specific ECM-associated features can condition specific gene expression and signaling pathways leading to the activation of the EMT program as well as the response to TGF-β1 [12]. Overall, these data demonstrated that 3D-specific ECM scaffolds are able to reproduce the interaction between resident liver cells and the surrounding ECM microenvironment and could play a crucial role in identifying new and more specific therapeutic targets.

In reason of the complexity in the development and progression of HCC, a rational analysis of the effect of disease-specific human liver ECM on co-cultures of HSC and/or HCC cells and their response to anti-fibrotic or anti-cancer drug therapy would certainly increase the current knowledge. Along these lines, the main objective of this study was to optimize and validate a 3D in vitro co-culture model using human healthy and cirrhotic liver 3D ECM scaffolds to evaluate a new platform for drug targeting and biomarkers identification.

## 2. Materials and Methods

### 2.1. Decellularization Protocols

Healthy human livers were retrieved for transplantation but judged unsuitable due to prolonged cold ischemic time, the presence of extra-hepatic cancer, or other significant extra-hepatic diseases in donors or recipients. Cirrhotic liver was explanted from a patient with alcoholic cirrhosis. Histological examination was performed to establish healthy and cirrhotic liver. Healthy liver was defined if there was no evidence of fibrosis and fat accumulation [12,21]. Optimization and characterization of the decellularized protocol were demonstrated in previous studies [12,21]. DNA and cell removal of decellularized liver scaffolds were examined by histology (H&E) and DNA quantification. Livers were obtained after approval by the UCL Royal Free BioBank Ethical Review Committee (NRES Rec Reference: 11/WA/0077). Informed consent was obtained for each donor and confirmed via the NHSBT ODT organ retrieval pathway [18,21]. Healthy and cirrhotic livers were cut into 125 mm^3^ cubes and stored at −80 °C until future use, as described previously [12]. Before decellularization, liver cubes were thawed in a water bath at 37 °C for 45 min, followed by the addition of 1% phosphate-buffered saline (PBS) and incubated for another 15 min. Liver cubes were transferred in the decellularization solution and placed in a TissueLyser II (QIAGEN). Details of the decellularization protocol, including frequency of oscillation, number of agitation cycles, and the solution for healthy and cirrhotic scaffolds are shown in Supplementary Table S1.

### 2.2. Cell Lines, Culture Conditions and Cell Treatment

HepG2, a human hepatoblastoma cell line [22], was purchased from ATCC (Manassas, VA, USA) and the LX2, a human Hepatic Stellate Cell line [23,24,25], was kindly provided by Professor Scott Friedman (Division of Liver Diseases, Icahn School of Medicine, Mount Sinai, New York, NY, USA). Both cell types were cultured in Iscove’s Modified Dulbecco’s Medium (IMDM, Gibco™, London, UK) supplemented with 10% fetal bovine serum (FBS), Penicillin-Streptomycin Gibco™ (1:100), 0.1 mM/L non-essential amino acids, 1.0 mM/L sodium pyruvate (all Gibco™, London, UK), at 37 °C and 5% CO_2_, as previously described [21]. Complete culture medium was changed every 3 days and cells were sub-cultured upon 70–80% confluence.

Two pharmacological agents were employed in this study to provide proof of concepts for the proposed 3D culture platforms. Sorafenib, which is still a first-line treatment for HCC, was employed as anti-fibrotic agent at a dose of 7 µM previously shown to exert anti-fibrotic effects in 3D spheroids co-cultures of LX2-HepG2 [26]. Therefore, LX2 and HepG2 cells repopulating the decellularized human liver scaffolds were treated with TGF-β1 (5 ng/mL) and/or Sorafenib (7 μM) for 48 h to test the anti-fibrotic effect of Sorafenib in this culture system.

Regorafenib, an oral multikinase inhibitor, currently recommended as second-line treatment for HCC in patient who progressed after Sorafenib treatment, was instead employed as an anti-cancer agent [3,27]. Thus, dose-dependent experiments were performed with Regorafenib (10 µM up to 40 µM) to establish the optimal dose concentration in 2D and 3D cultures. Thereafter, co-cultures repopulating the human liver scaffolds were exposed to TGF-β1 (5 ng/mL) and/or Regorafenib (16.6 μM) for 6 days for anti-cancer testing. Four replicates were performed in each condition.

### 2.3. Repopulation and Culturing of Engineered Human Liver

Decellularized liver scaffolds were sterilized using 1.5 mL of 0.1% peracetic acid (Sigma-Aldrich, Gillingham, UK) in 4% ethanol (Fisher Chemical, Loughborough, UK) for 45 min and 1.5 mL of sterile 1X Hank’s Balanced Salt Solution (HBSS, Gibco^TM^, Fisher Scientific, Loughborough, UK) for 30 min in an orbital shaker (Orbit M60, Labnet International, Inc., Edison, NJ, USA) at 500 rpm. Sterilized liver scaffolds were placed in a 48-well plate with complete culture medium and placed in the incubator for 24 h before repopulation.

For mono-cultures, cells were re-suspended at a concentration of 25 × 10^4^ cells in 20 µL and scaffolds, placed in a 96-well plate, and were repopulated with cells using the drop-on technique [12]. In the sequential co-culture (SeqCC), first LX2 cells were seeded for 1 week followed by an additional repopulation with HepG2 cells. In the simultaneous co-culture (SimCC), LX2 and HepG2 cell suspensions were mixed at a ratio of 1:1 (50 × 10^4^ cells in 20 µL) before repopulation. Seeded scaffolds were placed in a humidified incubator at 37 °C and 5% CO_2_ for 2 h followed by the addition of 140 µL of complete culture medium in each well (day 0). After 24 h of incubation, scaffolds were transferred to a 48-well plate and the culture medium was changed at day 1 and then every 3 days until day 13.

### 2.4. Histology and Immunostaining Analysis

Samples were fixed in 10% formalin for at least 24 h at room temperature (RT), processed and embedded in paraffin. Tissues were cut into 4 μm thick sections, dewaxed in xylene, and rehydrated with graded industrial denatured alcohol before staining as previously described [18,21]. Histology was performed by staining the tissue sections with Harris’s Hematoxylin and Eosin (H&E) (Leica, Wetzlar, Germany), and Picro-Sirius Red (SR) solutions (Hopkin and Williams) (BDH Chemicals Ltd., Cellpath Ltd., Newtown, UK). When performing immunohistochemistry, antigen retrieval was achieved with sodium citrate buffer (pH 6.0) in a microwave (640 W) for 10 min. Nonspecific binding was blocked with normal horse serum (VECTASTAIN^®^, Vector Laboratories, Peterborough, UK) for 5 min at RT. Sections were incubated for 1 h at RT with primary antibodies (see Supplementary Table S2). After washing, sections were incubated with pre-diluted biotinylated pan-specific universal secondary antibody (VECTASTAIN^®^, Vector Laboratories, Peterborough, UK) for 30 min. Primary antibodies were detected with the Novolink™ Kit (Novocastra RE7280-K, Newcastle, UK). Slides were counterstained with hematoxylin, dehydrated, cleared, and mounted. Images were captured using the Axiocam IcC5.

### 2.5. Viability Assessment

Cellular viability was evaluated using Prestoblue reagent according to the manufacturer’s instructions (Invitrogen, UK). Briefly, the Prestoblue solution (100 μL) was mixed with complete culture medium (900 μL) and added to each well. Scaffolds were incubated for 1.5 h at 37 °C and protected from direct light. Absorbance values were measured by microplate spectrophotometer (Fluorostar^®^ Omega, BMG LABTECH, Ortenberg, Germany) at wavelengths 530 nm excitation and 620 nm emission.

### 2.6. Gene Expression Analysis

RNA extraction was performed using the RNeasy mini kit (QIAGEN, Manchester, UK). Purity and RNA concentration were measured with a Nanodrop spectrophotometer (Thermo Scientific, Waltham, MA, USA). One microgram of RNA from each sample was reverse-transcribed with random primers and MultiScribe RT enzyme (Applied Biosystems, UK). Gene expression was measured by quantitative real-time polymerase chain reaction (qPCR) using Taqman gene assays (Supplementary Table S3) and a 7500 Fast Real-Time PCR System (Applied Biosystems). Levels of gene expression were calculated by the comparative CT method [28] using glyceraldehyde 3-phosphate dehydrogenase (GAPDH) as a reference gene.

### 2.7. Protein Extraction and Quantification

Bioengineered scaffolds were washed with 1X PBS and lysed with radio-immunoprecipitation assay (RIPA) buffer (20 mM Tris-HCL pH 7.6, 150 mM NaCl, 5 mM EDTA, 1% nonyl phenoxypolyethoxylethanol, 1mM phenylmethylsulfonyl fluoride, 1X Protease Inhibitors Mix, 1 mM Na3VO4, and 1 mM NaF) using 5 mm stainless steel beads (QIAGEN). Tubes were agitated for 5 min at full speed (50 cycles per second) using the TissueLyser (QIAGEN). Protein quantification was measured using the micro-bicinchoninic (BCA^TM^) Assay Kit (Thermo Scientific) according to the manufacturer’s protocol and samples were stored at −80 °C for further analysis.

### 2.8. Western Blot

Protein analysis was performed as previously described [29]. Briefly, to separate proteins, 20 μg of protein lysate of each sample was loaded onto sodium dodecyl sulfate-polyacrylamide gel electrophoresis (SDS-PAGE) using commercially available 4–12% SurePAGE^TM^, Bis-Tris gels (Genscript^®^, Piscataway, NJ, USA). Proteins were transferred to polyvinylidene fluoride (PVDF) membrane (Immobilon-P Transfer Membranes, Millipore^TM^, Billerica, MA, USA) with a transfer buffer containing 25 mM Tris, 192 mM Glycine, and 10% Methanol at 100 V for 75 min. The membranes were stained with Ponceau S solution (0.1% (*w*/*v*) in 5% acetic acid, Sigma-Aldrich) to verify efficient transfer. After blocking with 5% bovine serum albumin (BSA) for 1 h, membranes were probed overnight at 4 °C with primary antibodies and 1 h at RT with corresponding secondary antibodies. Immunoreactive bands were detected by enhanced chemiluminescence with protein A-horseradish peroxidase and the Supersignal^®^ West Pico Chemiluminescent Substrate (Thermo Scientific). Equal protein loading was confirmed by analyzing the expression of housekeeping proteins GAPDH or histone H3. Details of primary and secondary antibodies used in this study are listed in Supplementary Table S4.

### 2.9. Protein Secretion Analysis

Secreted pro-collagen 1a1 levels in the culture media were detected by using the Human Pro-Collagen 1 alpha 1 SimpleStep ELISA^®^ Kit (ab210966, Abcam, Cambridge, UK) according to the manufacturer’s protocol and as described previously [18].

### 2.10. Flow Cytometry Analysis

The immunophenotypic characterization of LX2 and HepG2 cells in single and co-cultures was identified by flow cytometry and by analyzing the epithelial cellular adhesion molecule EPCAM protein (antibody CD326, BD Bioscience, San Diego, CA, USA) which is strongly expressed by HepG2 cells. Briefly, after 13 days of culture cells were extracted from the scaffolds by incubation in a collagenase type IV solution at 1 mg/mL (Sigma-Aldrich, Gillingham, UK, C5138) for 30 min at 37 °C. The cell suspension was then passed through a 35 µm nylon cell strainer and chased with a rinse of an additional 5 mL of staining buffer. Cells were incubated for 20 min to block non-specific binding (Human Fc Block, BD Bioscience, San Diego, CA, USA, 564219). Cells were stained with an antibody against the surface marker EPCAM conjugated to BD Horizon™ BV421. Cells were analyzed by fluorescence-activated cell sorting (FACS) using a BD Fortessa II instrument (BD Biosciences, San Jose, CA, USA). Samples were examined by side scatter area (SSC-A) versus forward scatter area (FSC-A), then using forward scatter height (FSC-H) versus FSC-A to select single cells, eliminating collagen debris and clumped cells from the analysis. Single cells were sub-gated using EPCAM antibody and subsequently, HepG2 and LX2 were discriminated by positivity or absence of its expression, respectively. After acquisition, the data were exported and analyzed using FlowJo (Ashland, OR, USA).

### 2.11. Statistical Analysis

Values are presented as the mean ± standard deviation (SD). Statistical significance was analyzed using unpaired *t*-test, non-parametric test of one-way ANOVA with Graph Pad Prism software. A two-tailed *p*-value < 0.05 was considered statistically significant.

## 3. Results

### 3.1. Optimization of LX2-HepG2 Cell Co-Culture Model in Human Liver 3D Scaffolds

In the first set of experiments, we investigated whether a 3D co-culture system of LX2 and HepG2 cells could affect cell-cell specific functions compared to 3D mono-cultures. Two reseeding co-culture protocols were tested: (1) a simultaneous repopulation (SimCC) of LX2 cells with HepG2 cells, and (2) a sequential repopulation (SeqCC) in which LX2 cells were first reseeded followed by the introduction of HepG2 cells at day 7. The sequential co-culture protocol was based on the assumption that LX2 cells would favor a better engraftment of HepG2 cells inside the scaffold by remodeling the scaffold ECM when engrafting and thus promoting a more efficient migration of HepG2 in the 3D structure.

**Figure 1:**
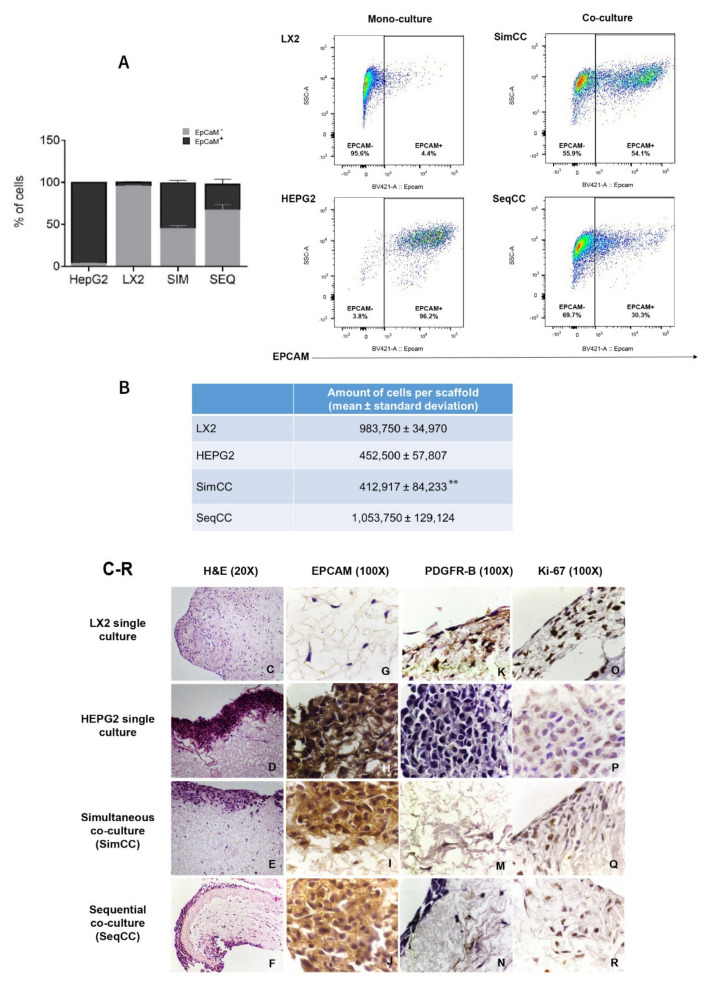
Localization and fluorescence-activated cell sorting (FACS) analysis of LX2 and HepG2 cells in single and co-cultures in 3D liver scaffolds. (**A**) Percentage of LX2 and HepG2 cells engrafted in healthy scaffolds in single cultures of LX2 or HepG2 cells, and co-cultures SimCC and SeqCC as determined by Flow cytometry analysis using EPCAM antibody. (**B**) Quantification of engrafted cells in single-cell cultures of LX2, HepG2, and co-cultures SimCC and SeqCC in healthy 3D liver scaffolds (** *p* ≤ 0.01 SimCC compared to SeqCC). (**C**–**F**) Hematoxylin and Eosin staining of LX2 and HepG2 repopulating 3D ECM healthy liver scaffolds showed LX2 cells diffusely spread inside the scaffold, while HepG2 cells engrafted at the periphery of the 3D scaffold (20X magnification). (**G**–**J**) Distribution of each cell type in single cultures, simultaneous co-culture (SimCC), and sequential co-culture (SeqCC) confirmed by epithelial cell marker EPCAM and (**K**–**N**) LX2 cell marker PDGFR-β immunohistochemistry (100× magnification). (**O**–**R**) Cell proliferation was detected by performing Ki-67 immunohistochemistry (100× magnification).

First, FACS analysis was employed to determine the proportion/ratio of LX2 and HepG2 engrafting the healthy 3D liver scaffolds in single and co-culture models (SimCC and SeqCC) (Figure 1A). The immunophenotypic characterization of LX2 and HepG2 cells in co-cultures was identified by analyzing EPCAM expression. As demonstrated in Figure 1A, the proportion of EPCAM-positive cells was 96.2 ± 0.7% in HepG2 mono-culture versus 4.4 ± 1.2% in LX2 mono-culture. Importantly, SimCC showed an equal portion between HepG2 and LX2 cells (54.1 ± 6.2%), whereas SeqCC contained more LX2 cells than HepG2 with a 30.3 ± 12.2% EPCAM positive cells. Indeed, quantifying the total number of engrafted cells in all conditions showed that in LX2 mono-cultures the total number was 9.8 × 10^5^ ± 0.3 mean ± sd and HepG2 mono-culture 4.5 × 10^5^ ± 0.6 mean ± sd (Figure 1B). Moreover, when comparing both co-culture systems the total number of cells engrafting the 3D scaffolds in SimCC was less than SeqCC i.e., 4.1 ± 0.8 (×10^5^) versus 10.5 ± 1.3 (×10^5^) (** *p*≤ 0.01) (Figure 1B) with more LX2 cells present than HepG2 cells as shown in Figure 1A. These data indicated that in the SeqCC co-culture model, during the first 7 days, LX2 cells proliferate which was then followed by the repopulation of HepG2 cells resulting in more LX2 cells than HepG2 cells. In contrast, SimCC demonstrated a ratio of 1:1 of LX2 and HepG2 cells repopulating the 3D liver scaffolds.

Accordingly, as shown with H&E staining (Figure 1C–F), mono-cultures of LX2 cells diffusely repopulated the scaffolds (Figure 1C), whereas mono-cultures of HepG2 cells showed an engraftment limited to the margins of the scaffolds (Figure 1D). In co-cultures, the precise location of both cell types was confirmed by immunohistochemistry staining for the HepG2 epithelial cell marker EPCAM (Figure 1G–J) and the HSC activation marker PDGFR-β (Figure 1K–N). Further, both mono-cultures and co-cultures showed positive stained Ki-67 cells indicating cell proliferation (Figure 1O–R).

### 3.2. Characterization of Cell Phenotype in Co-Cultures Versus Single Cultures in 3D ECM Scaffolds

**Figure 2:**
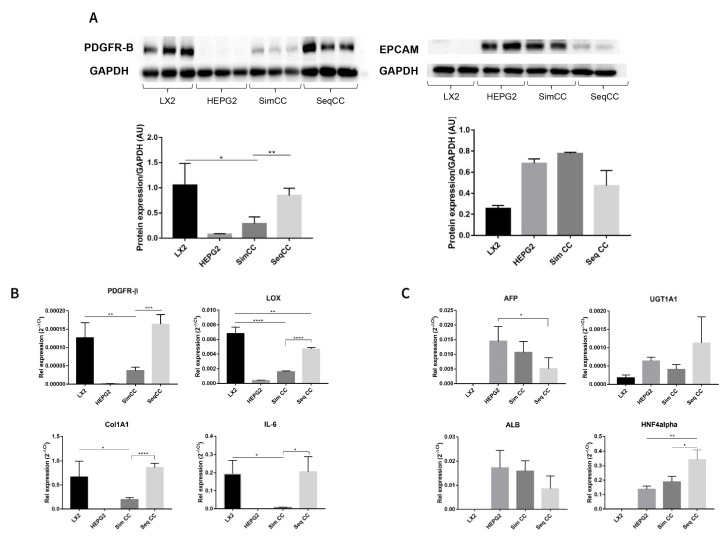
Characterization of mono-culture and co-culture of LX2 and HepG2 cells in 3D human healthy liver scaffolds. (**A**) Western blot and densitometry analysis of PDGFR-β and EPCAM protein expression of LX2 and HepG2 cells in single culture, simultaneous (SimCC), and sequential co-culture (SeqCC) (AU arbitrary units). (**B**) mRNA expression of genes related to pro-fibrogenesis and inflammation. (**C**) mRNA expression of genes related to hepatocellular carcinoma cell phenotypes and hepatocyte-specific functions. (*n* = 4 scaffolds per condition, * *p* ≤ 0.05, ** *p* ≤ 0.01, *** *p* ≤ 0.001, and **** *p* ≤ 0.0001). (Figure S1: Whole blot showing all the bands with all molecular weight markers on the Western).

In this set of experiments, cell-specific markers were evaluated to further identify the effect of single cultures versus co-cultures and the effect of repopulation on 3D ECM scaffolds. PDGFR-β protein expression showed a significant downregulation in SimCC compared to LX2 mono-cultures (* *p* ≤ 0.05). In contrast, PDGFR-β protein expression showed significant upregulation in SeqCC versus SimCC (** *p* < 0.01) further confirming that the majority of cells in SeqCC were LX2 cells as suggested by FACS analysis (Figure 1A,B). HepG2 cells grown in mono-culture and SimCC showed a similar tendency towards increased EPCAM protein expression while those in SeqCC showed a trend towards downregulation of EPCAM expression compared to SimCC (Figure 2A), confirming the FACS analysis data that demonstrated more LX2 cells than HepG2 cells in SeqCC (Figure 1A).

The same pattern was observed at the mRNA expression level of *PDGFR-β* (** *p* ≤ 0.01), *COL1A1* (* *p* ≤ 0.05), *LOX* (**** *p* ≤ 0.0001) and *IL-6* (* *p* ≤ 0.05) which showed to be significantly down-regulated in SimCC compared to mono-cultures of LX2 cells (Figure 2B). As also suggested by FACS analysis, the majority of cells in SeqCC were LX2 cells (Figure 1B), as confirmed by the significant upregulation in *PDGFR-β* (*** *p* ≤ 0.001), *COL1A1* (**** *p* ≤ 0.0001), *LOX* (**** *p* ≤ 0.0001) and *IL-6* expression (* *p* ≤ 0.05) in comparison to SimCC (Figure 2B). Gene expression of *AFP, ALB*, *UGT1A1*, and *HNF4A* showed similar mRNA levels when comparing mono-cultures of HepG2 with SimCC (Figure 2C). *AFP* gene expression was significantly downregulated in SeqCC in comparison to mono-cultures of HepG2 (* *p* ≤ 0.05), and *ALB* showed a tendency to decrease in SeqCC versus mono-cultures and SimCC. *HNF4A* mRNA expression was significantly upregulated in SeqCC versus SimCC (* *p* ≤ 0.05) and mono-cultures (** *p* ≤ 0.01).

In conclusion, the above data support the observation that SimCC represents a more accurate co-culture system to further investigate the effect of TGF-β1 in 3D co-culture systems. Therefore, the following sets of experiments were performed using simultaneous co-cultures (SimCC).

### 3.3. Response to TGF-β1 Treatment in Single and Simultaneous Co-Cultures in Healthy and Cirrhotic 3D Scaffolds

In the next set of experiments, cells were engrafted in healthy liver 3D scaffolds to investigate possible differences between mono-cultures of LX2 cells and HepG2 cells and the simultaneous co-culture (SimCC), upon TGF-β1exposure known to affect liver fibrogenesis and tumor cell differentiation. Furthermore, in a separate experiment, cells were engrafted in cirrhotic liver 3D scaffolds to investigate the possible impact of disease-derived ECM on cell behavior.

**Figure 3:**
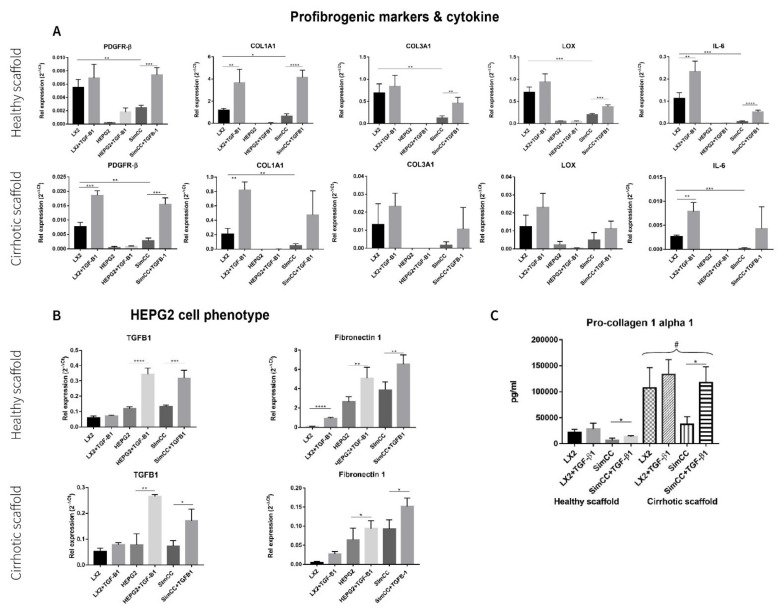
Response to TGF-β1 exposure of LX2 and HepG2 cells mono-culture and co-culture in 3D healthy and cirrhotic ECM liver scaffolds. (**A**) Gene expression related to pro-fibrogenesis and cytokines in different culture conditions tested. (**B**) Changes in HepG2 gene expression related with hepatocellular carcinoma cell progression, *n* = 4 scaffolds per condition. (**C**) LX2 cells pro-collagen 1a1 secretion as single culture and simultaneously co-cultured (CC) with HepG2 cells, with or without TGF-β1 (5 ng/mL) exposure (# = *p* ≤ 0.01 for each condition in 3D healthy scaffold versus 3D cirrhotic scaffold) (* *p* ≤ 0.05, ** *p* ≤ 0.01, *** *p* ≤ 0.001, and **** *p* ≤ 0.0001).

First, the mRNA expression levels for profibrogenic genes were investigated in healthy 3D scaffolds. All genes under investigation showed significant downregulation in SimCC versus LX2 mono-cultures grown in 3D healthy scaffolds further confirming the result shown in Figure 2, whereas *PDGFR-β* (*** *p* ≤ 0.001), *COL1A1* (**** *p* ≤ 0.0001), *COL3A1* (** *p* ≤ 0.01), *LOX* (*** *p* ≤ 0.001), and *IL-6* (** *p* ≤ 0.0001) showed to be significantly upregulated in SimCC exposed to TGF-β1 in comparison to SimCC without TGF-β1 treatment (Figure 3A).

Next, profibrogenic gene expression was evaluated in bioengineered cirrhotic 3D scaffolds and showed a significant upregulation in mRNA expression for *PDGFR-β* (*** *p* ≤ 0.001), *COL1A1* (** *p* < 0.01), and IL-6 (** *p* ≤ 0.01) in LX2 cells treated with TGF-β1 versus LX2 without TGF-β1 exposure (Figure 3A). In contrast to SimCC cultured in healthy 3D scaffolds, only *PDGFR-β* mRNA expression (*** *p* ≤ 0.001) was significantly upregulated in SimCC in 3D cirrhotic scaffolds upon TGF-β1 treatment versus non-treated SimCC, suggesting an ECM-specific effect.

Furthermore, the effect of TGF-β1 exposure on HepG2 cells cultured in mono-culture and SimCC in 3D healthy and cirrhotic scaffolds showed a significant upregulation in mRNA gene expression of *TGF-β1* (* *p* ≤ 0.05) and *Fibronectin-1* (* *p* ≤ 0.05) in mono-cultures treated with TGF-β1 compared to mono-cultures of HepG2 without TGF-β1 treatment and in SimCC cultures exposed to TGF-β1 versus SimCC without TGF-β1 treatment (Figure 3B).

To further investigate the effect of TGF-β1-induced expression on *COL1A1* expression, a key pro-fibrogenic ECM factor, LX2 mono-culture and SimCC were grown simultaneously in healthy and cirrhotic 3D scaffolds. The secretion of pro-collagen1a1 showed a significant upregulation in all conditions when LX2 cells were engrafted in cirrhotic 3D scaffolds versus healthy 3D scaffolds (# *p* ≤ 0.01) (Figure 3C). This suggested that tissue-specific ECM can induce progression of liver fibrosis as shown in cirrhotic 3D scaffolds versus healthy 3D scaffolds. Further, LX2 cells exposed to TGF-β1 for 48 h and grown in SimCC significantly secreted more pro-collagen1a1 protein compared to those in SimCC without TGF-β1 exposure in both 3D healthy (* *p* ≤ 0.05) and cirrhotic liver scaffolds (* *p* ≤ 0.05) (Figure 3C). Overall, these data demonstrate significant differences in protein and gene expression between cells grown in mono-culture versus SimCC and when engrafted in healthy 3D scaffolds versus 3D cirrhotic scaffolds with or without TGF-β1 treatment.

### 3.4. Evaluating Anti-Fibrotic Drug Response of Sorafenib in LX2-HepG2 Co-Culture Model in 3D Liver Scaffolds

**Figure 4:**
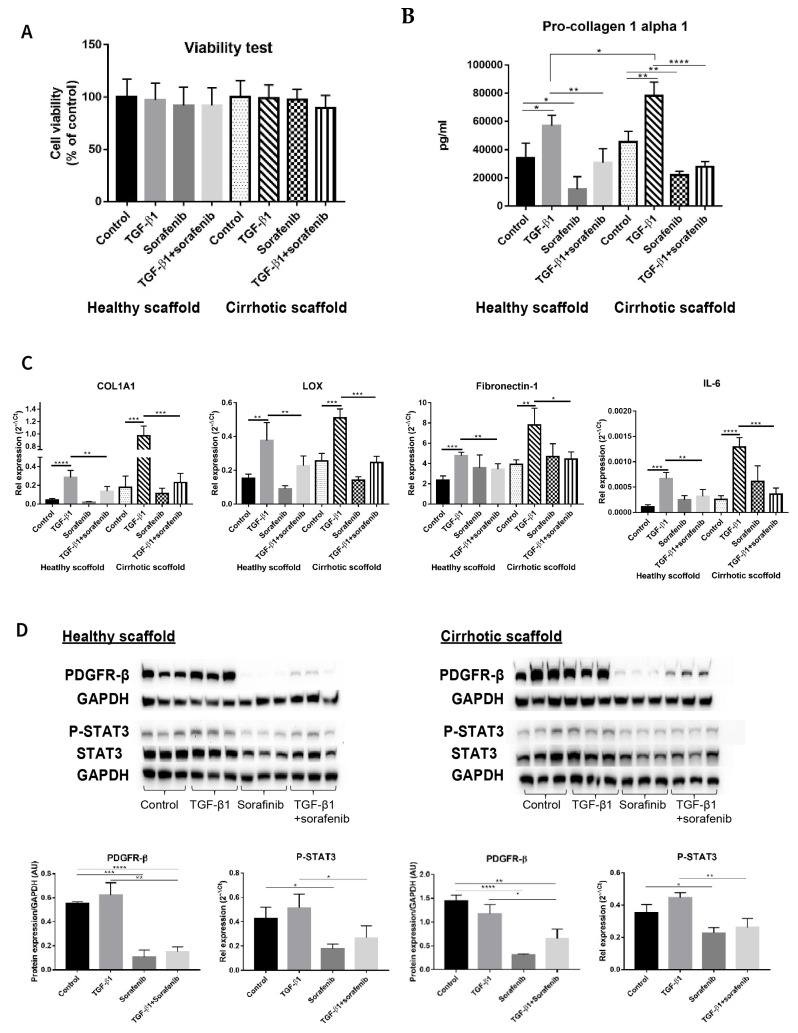
Validation of Sorafenib’s anti-fibrotic effect on LX2 -HepG2 co-culture model in 3D healthy and cirrhotic human liver scaffolds. (**A**) Viability response of LX2-HepG2 cells co-cultured in 3D ECM liver scaffolds to TGF-β1 5 ng/mL, Sorafenib 7 µM and combination therapy for 48 h. (**B**) Pro-collagen1 α1 secretion in LX2-HepG2 cells co-cultured in 3D ECM scaffolds exposed to TGF-β1 (5 ng/mL), Sorafenib (7 µM), and combination therapy for 48 h. (**C**) Western blot and densitometry analysis of PDGFR-β, phosphorylated STAT3, and STAT3 expression from LX2/HepG2 cells co-cultured and treated with TGF-β1 (5 ng/mL), Sorafenib (7 µM), and combination therapy for 48 h in 3D ECM scaffolds using GAPDH as a loading control. (**D**) Gene expression of pro-fibrogenic markers of LX2/HepG2 cells co-cultured and treated with TGF-β1 (5 ng/mL), Sorafenib (7 µM), and combination therapy for 48 h in 3D ECM scaffolds. (*n* = 4 scaffolds per condition, * *p* ≤ 0.05, ** *p* ≤ 0.01, *** *p* ≤ 0.001, and **** *p* ≤ 0.0001). (Figure S1: Whole blot showing all the bands with all molecular weight markers on the Western).

Although Sorafenib is known as a first level anti-cancer drug for patients with advanced tumors [3,30,31], it is also known to have anti-fibrotic effects in in vitro and in vivo models [32,33,34] and its targets have been well elucidated in several reports [32,35,36]. Thus, simultaneous co-cultures of LX2 and HepG2 (SimCC) were treated with Sorafenib (7 µM) as was previously shown to have anti-fibrotic effects in LX2-HepG2 co-cultures in 3D spheroids [26] with or without TGF-β1 (5 ng/mL) for 48 h. First, cell viability was quantified to exclude Sorafenib’s possible anti-cancer effect, i.e., apoptosis-inducing effect, as in this experiment Sorafenib’s anti-fibrotic effect will be investigated. As shown in Figure 4A, exposure to Sorafenib of co-cultures in both types of scaffolds, with or without TGF-β1, did not affect cell viability when compared to non-treated control conditions thus excluding Sorafenib’s apoptotic-inducing effect.

Importantly, pro-collagen1a1 protein secretion was decreased in co-cultures treated with Sorafenib plus TGF-β1 compared to cells treated with TGF-β1 in healthy (** *p* ≤ 0.01) and cirrhotic liver 3D scaffolds (**** *p* ≤ 0.0001) (Figure 4B). Furthermore, Sorafenib significantly reduced TGF-β1-induced upregulation of *COL1A1*, *LOX*, *Fibronectin-1*, and *IL-6* mRNA expression in LX2-HepG2 co-cultures both engrafted in healthy (** *p* ≤ 0.01) and cirrhotic liver 3D scaffolds (*** *p* ≤ 0.001 for *COL1A1*, *LOX*, and *IL-6* and * *p* < 0.05 for *Fibronectin-1*) (Figure 4C).

Protein analysis showed that Sorafenib treatment reduced PDGFR-β expression in co-cultures versus control co-cultures in healthy 3D scaffolds (*** *p* < 0.001) and in cirrhotic scaffolds (**** *p* ≤ 0.0001) indicating Sorafenib’s potential anti-proliferative/anti-fibrogenic effect. Moreover, Sorafenib in combination with TGF-β1, significantly inhibited TGF-β1-induced upregulation of PDGFR-β and phosphorylation of STAT3 protein expression in comparison to TGF-β1-exposed co-cultures in both types of scaffolds (** *p* ≤ 0.01, * *p* ≤ 0.05) (Figure 4D).

### 3.5. Chemoresistance in LX2-HepG2 Co-Culture System in 3D Healthy and Cirrhotic Liver Scaffolds

**Figure 5:**
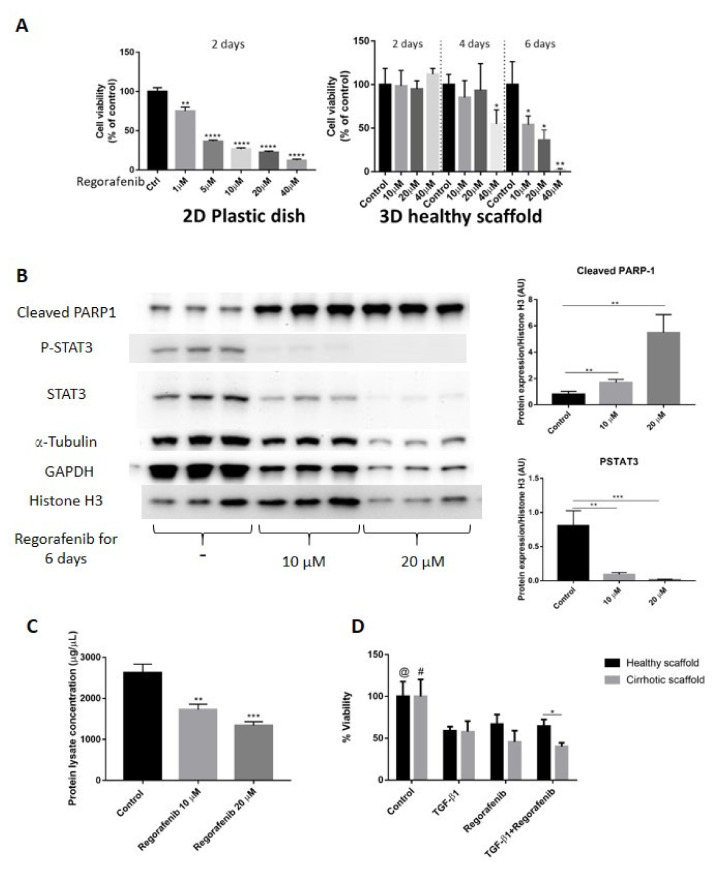
Validation of Regorafenib’s anti-cancer effect on LX2 and HepG2 cell co-culture model in 3D healthy and cirrhotic human liver scaffolds. (**A**) Viability response of LX2/HepG2 cells co-cultured in 2D plastic dish and 3D healthy ECM liver scaffold to Regorafenib. Cells were exposed with a dose escalation and time escalation of Regorafenib (0, 1, 5, 10, 20, and 40 µM). (**B**) Western blot and densitometry analysis of cleaved PARP-1, P-STAT3, and STAT3 expression in co-culture model in 3D healthy scaffolds treated with Regorafenib (0, 10, and 20 µM) for 6 days. The α-Tubulin and GAPDH were used as housekeeping proteins. (*n* = 4 scaffolds per condition * *p* ≤ 0.05, ** *p* ≤ 0.01, *** *p* ≤ 0.001, and **** *p* ≤ 0.0001). (**C**) Total protein lysate concentration of LX2/HepG2 cells seeded in 3D healthy control and treated with regorafenib 10 and 20 µM. (**D**) Viability response of LX2/HepG2 cells grown in 3D healthy and cirrhotic liver scaffolds to TGF-β1 5 ng/mL, Regorafenib 16.6 µM, and combination therapy for 6 days. @ indicates *p* ≤ 0.05 for control vs. other conditions in healthy scaffolds. # indicates *p* ≤ 0.05 for control vs. other conditions in cirrhotic scaffolds. (Figure S1: Whole blot showing all the bands with all molecular weight markers on the Western).

In this set of experiments, we validated the co-cultures and 3D ECM scaffolds as platforms to investigate the anti-cancer effects by evaluating Regorafenib, an oral multikinase inhibitor, which is currently recommended as a second-line anti-cancer treatment for patients with advanced HCC who progressed after Sorafenib treatment [3,27]. First, we determined Regorafenib’s concentration required to inhibit 50% HepG2-LX2 cell viability (IC_50_) in 2D cell cultures and co-cultures engrafting healthy 3D scaffolds. To calculate IC_50_, the inhibitory concentration against cell viability was plotted with Graph Pad Prism software, followed by using the dose–response-inhibition equation under nonlinear regression analysis to identify IC_50_ which was calculated to be 16.6 µM. Important, we demonstrate that to obtain Regorafenib’s IC_50_, a four-fold higher dose of Regorafenib and a two-fold increase in exposure time was needed in LX2-HepG2 co-cultures engrafted in 3D scaffolds compared to 2D cultures (16.6 μM for 6 days in 3D versus 3.6 μM for 2 days in 2D) (Figure 5A). Next, co-cultures were engrafted in 3D healthy scaffolds and treated with Regorafenib (16.6 µM for 6 days). Regorafenib’s anti-cancer effect, i.e., induction of apoptosis was further demonstrated at the protein level, by activation of PARP-1 cleavage protein expression in a dose-dependent manner when compared with control co-culture cells (Figure 5B). Phosphorylated STAT3 has been reported to modulate Bcl-2 gene expression involved in the anti-apoptotic response of HCC cells, resulting in caspase-3 activation and increased PARP cleavage [37]. Indeed, our data demonstrated that Regorafenib’s-induced PARP-1 expression coincided with a reduction in phosphorylation of STAT3 (P-STAT3) and STAT3 protein expression in Regorafenib exposed cells versus control non-treated co-cultured cells. Regorafenib downregulated both α-tubulin and GAPDH protein expression in a dose-dependent way, as previously demonstrated [38]. Furthermore, total protein quantification indicated that the concentrations of total protein lysate obtained from co-cultures engrafting healthy 3D scaffolds and treated with Regorafenib (10–20 µM for 6 days) were lower than in control co-cultures in 3D healthy liver scaffolds (** *p* ≤ 0.01) (Figure 5C), further suggesting Regorafenib’s apoptotic effect. Overall, these data demonstrated that Regorafenib induced apoptosis in LX2-HepG2 co-cultures engrafting 3D healthy liver scaffolds.

Next, we investigated whether specific ECM could have an impact on Regorafenib’s efficacy or could induce chemoresistance in bioengineered co-cultures exposed to TGF-β1. Thus, co-cultures were treated with Regorafenib (16.6 µM) with/without TGF-β1 (5 ng/mL) for 6 days, and cell viability was compared between HepG2-LX2 co-cultured in healthy versus cirrhotic 3D scaffolds. Regorafenib treatment with or without TGF-β1 reduced cell viability compared to control non-treated co-cultures in both 3D healthy scaffold (@ *p* ≤ 0.05) and 3D cirrhotic scaffolds (# *p* ≤ 0.05) (Figure 5D). Importantly, co-cultures repopulating the cirrhotic 3D scaffolds exposed to Regorafenib and TGF-β1 showed less viability in comparison to bioengineered healthy 3D scaffolds (* *p* ≤ 0.05) as was further shown by H&E staining (Supplementary Figure S2).

Overall, these sets of data demonstrated differences in chemoresistance upon Regorafenib exposure between 2D co-cultures versus co-cultures in 3D liver healthy and cirrhotic scaffolds (Figure 5A,D).

### 3.6. Regorafenib Inhibits TGF-β1-Induced EMT and STAT3 Phosphorylation in 3D Human Liver Scaffold Model

**Figure 6:**
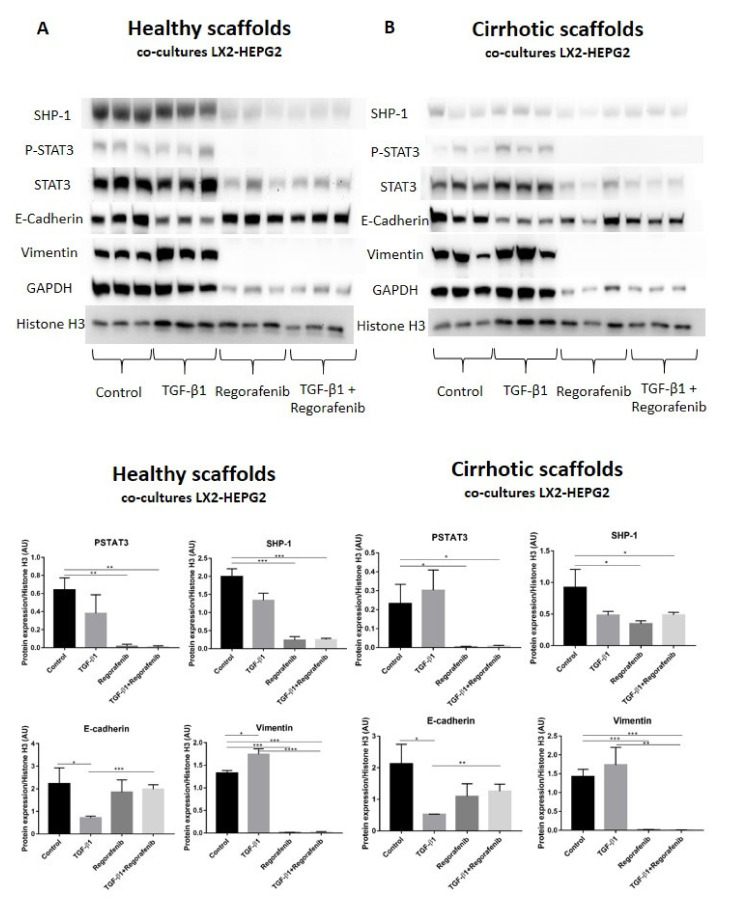
Protein expression of the LX2 and HepG2 cell co-cultures treated with TGF-β1 and/or Regorafenib in healthy and cirrhotic liver 3D scaffolds. Western blot and densitometry analysis of SHP-1, P-STAT3, STAT3, E-cadherin, Vimentin, and GAPDH protein expression of LX2 and HepG2 cells co-cultured in 3D healthy (**A**) and cirrhotic scaffolds (**B**) following TGF-β1 5 ng/mL with/without Regorafenib 16.6 µM exposure for 6 days (*n* = 4 scaffolds per condition, * *p* ≤ 0.05, ** *p* ≤ 0.01, *** *p* ≤ 0.001, and **** *p* ≤ 0.0001). (Figure S1: Whole blot showing all the bands with all molecular weight markers on the Western).

A recent study demonstrated that phosphorylated STAT3 is a positive regulator of TGF-β1-induced EMT in an in vivo HCC model and in liver tissue from HCC patients [39]. In contrast, Src homology region 2 (SH2) domain-containing phosphatase 1 (SHP-1) is a negative regulator of EMT in HCC cells by downregulating P-STAT3 [40]. Thus, we investigated whether SHP-1 and P-STAT3 protein expression was involved in Regorafenib’s effect on reducing TGF-β1-induced EMT in the new co-culture system. Phosphorylation of STAT3 was not significantly changed upon TGF-β1 exposure in bioengineered co-cultures in healthy and cirrhotic 3D scaffolds in comparison to control cells (Figure 6A,B). In contrast, P-STAT3 was significantly downregulated in co-cultures exposed to Regorafenib with or without TGF-β1 versus control cells in healthy 3D scaffolds (** *p* ≤ 0.01) and cirrhotic 3D scaffolds * *p* ≤ 0.05). Important, also SHP-1 expression significantly decreased in co-cultures treated with Regorafenib with or without TGF-β1 versus control cells in healthy 3D scaffolds (*** *p* ≤ 0.001) and cirrhotic 3D scaffolds (* *p* ≤ 0.05). As both *p*-STAT3 and SHP-1 are known to affect TGF-β1-induced EMT in HCC, the expression of downstream proteins such as E-cadherin and vimentin was investigated. TGF-β1 exposure significantly downregulated E-cadherin protein expression in both healthy and 3D cirrhotic scaffolds (* *p* ≤ 0.05) in comparison to non-treated control co-cultures. This coincided with an increase in vimentin protein expression. Important, Regorafenib significantly reversed the TGF-β1-induced EMT as shown by increased E-cadherin expression in co-cultures exposed simultaneously to TGF-β1 and Regorafenib in healthy (*** *p* ≤ 0.001) and cirrhotic 3D scaffolds (** *p* ≤ 0.01) which coincided with a significant reduction in Vimentin protein expression (* *p* ≤ 0.05) in both 3D models (Figure 6A,B). These data indicated that Regorafenib inhibits TGF-β1-induced EMT.

**Figure 7:**
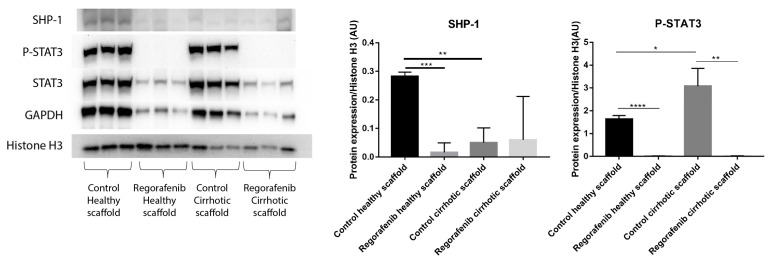
Protein expression of LX2-HepG2 co-cultures treated with Regorafenib compared to control in healthy and cirrhotic 3D scaffolds. Western blot and densitometry analysis of SHP-1, P-STAT3, STAT3 and GAPDH protein expression of LX2 and HepG2 cells co-cultured in healthy and cirrhotic 3D liver scaffolds treated with Regorafenib 16.6 µM for 6 days compared to internal controls (*n* = 4 scaffolds per condition, * *p* ≤ 0.05, ** *p* ≤ 0.01, *** *p* ≤ 0.001, and **** *p* ≤ 0.0001). (Figure S1: Whole blot showing all the bands with all molecular weight markers on the Western).

In a previous study, we demonstrated that the cirrhotic liver ECM has a unique ECM signature that affect protein and gene expression in HepG2 cells. Cells grown in the healthy and cirrhotic liver exhibited different cellular behavior [12]. Therefore, co-culture samples obtained from both healthy and cirrhotic 3D ECM scaffolds were run in parallel to investigate the effect of the healthy and cirrhotic 3D ECM scaffolds on co-cultures treated with or without Regorafenib (Figure 7). Phosphorylation of STAT3 was significantly induced when co-cultures were engrafted in cirrhotic 3D scaffolds versus healthy 3D scaffolds (* *p* ≤ 0.05). Nevertheless, the effect of tissue-specific ECM on P-STAT3 expression was abolished when co-cultures were treated with Regorafenib as demonstrated by a significant reduction in P-STAT3 in comparison to their internal control (** *p* ≤ 0.01) and this in both types of ECM 3D scaffolds (Figure 7). In contrast, SHP-1 protein expression was significantly downregulated in non-treated co-cultures engrafting 3D cirrhotic scaffolds versus non-treated co-cultures engrafting 3D healthy scaffolds (** *p* ≤ 0.01) indicating a possible specific ECM effect on SHP-1 protein expression. Regorafenib significantly reduced SHP-1 protein expression in co-cultures engrafting the 3D healthy scaffolds versus non-treated co-cultures engrafting 3D healthy scaffolds but not 3D cirrhotic scaffolds (Figure 7).

## 4. Discussion

In the present study, we optimized and validated an advanced 3D model by using human healthy and cirrhotic liver 3D ECM scaffolds engrafted with LX2 and HepG2 cells. We provide evidence that this model is able to better recapitulate the key cellular and molecular events leading to liver fibrosis and to mimic the complexity of the tumor microenvironment with stromal cells and cancer cells when compared to standard 2D culture systems. Accordingly, this 3D co-culture model could be applied for dual screening for anti-fibrotic and anti-cancer drugs. Moreover, we demonstrated that tissue-specific ECM can provide key insights into liver fibrosis and pro-cancerogenic TGFβ1-induced EMT in fibroblastic stromal cells and cancer cells, respectively.

We previously demonstrated that decellularized human liver 3D scaffolds retain the key features of their natural 3D microenvironment and vascular network in the absence of cellular and nuclear components [18,21] and that this system is a suitable platform for the study of tissue-specific and disease-specific cell–cell crosstalk and cell–ECM interactions [18,21].

In the current study, we optimized a model using LX2 and HepG2 cells in 3D tissue-specific and disease-specific ECM human liver scaffolds. LX2 is a cell line that preserves the in vivo characteristics of activated human HSC by expressing several key receptors and proteins involved in liver fibrosis and ECM remodeling [23,24,25]. HepG2 is a well-established human hepatoblastoma cell line and used for high-throughput drug screening [22,41]. Our results demonstrated that co-cultures of LX2-HepG2 cells in 3D ECM liver scaffolds could be used to investigate the HSC/HCC cell-crosstalk and to identify possible new anti-cancer targets. Further demonstrating the importance of using co-cultures for studying the crosstalk in the context of tumor–stromal cell behavior as we and others have shown [42,43].

An additional important finding of this study is the demonstration that the difference in ECM biochemical and biomechanical properties can affect resident cell behavior. It is known that the alteration in liver stiffness is an important key factor driving the progression of liver fibrosis and cancer [11,12]. Furthermore, mechanical stress exerted by the abnormal stiffness of the fibrotic microenvironment is important for HSC activation and transdifferentiation [44]. Recently, we demonstrated that decellularized 3D cirrhotic liver ECM scaffolds promoted HCC cells–EMT induction in single-cell cultures of HepG2 compared to decellularized 3D healthy liver scaffolds, unraveling the importance of tissue-specific ECM on cell behavior [12]. Previous studies reported that the 3D microenvironment of fibrotic liver promoted HCC cells progression and chemoresistance [43,44], and several studies demonstrated that ECM-producing stromal cells are able to induce drug resistance and tumor progression in HCC cells in a 3D spheroid co-culture model [1,2,14,23]. In the present study, we show that gene expression and protein secretion related to pro-fibrogenic and pro-cancerogenic progression were 1–3-fold upregulated when LX2 and HepG2 cells were grown in 3D cirrhotic scaffolds in comparison to 3D healthy scaffolds (Figure 4B,C). These data indicated that the stiffness of ECM, but also the specificity in ECM components [12], affects cellular response in both single and co-culture systems.

TGF-β1 is a central regulator involved in the pathogenesis of liver fibrosis/cirrhosis and HCC progression. The effect of TGF-β1 in the progression of HCC depends on the stage of tumor development with facilitation of cancer cell migration at a later stage [45,46]. Moreover, TGF-β1 exerts profound pro-fibrogenic effects on HSC which become an integral part of the tumor stroma [47,48]. Our results show that LX2-HepG2 co-cultures in 3D healthy and cirrhotic ECM scaffolds were able to respond to TGF-β1 exposure by increasing the secretion/mRNA expression in LX2 cells related to HSC activation (PDGFR-β) and ECM production (pro-collagen1a1, COL1A1, COL3A1, and LOX) in comparison to the mono-cultures. TGF-β1 treatment of HepG2 cells in both types of scaffolds, in either mono-culture or co-cultures, induced fibronectin-1 and TGF-β1 mRNA expression, suggesting HCC progression [49]. Overall, these results show that co-cultures of 3D liver ECM scaffolds can recapitulate cellular and molecular events upon TGF-β1 exposure leading to liver fibrosis and HCC progression.

Sorafenib is an oral multikinase inhibitor and standard treatment for patients with advanced stages of HCC. Recently, the anti-fibrotic effect of Sorafenib has been well documented by several in vitro and in vivo studies [32,33,34,36,50]. Accordingly, it was shown that Sorafenib treatment was able to ameliorate liver cirrhosis by reducing liver fibrosis, inflammation, angiogenesis, and portal hypertension in cirrhotic rats [51,52]. One important transcription factor in this process is STAT3 known to play a key role in liver fibrosis and tumor progression [53]. STAT3 activation promotes HSC survival, proliferation, and activation, contributing to liver fibrogenesis [54]. In this study, we demonstrated that Sorafenib, at an anti-fibrotic and non-toxic dose, reduced pro-collagen1a1 secretion, PDGFR-β protein expression, and COL1A1, LOX, Fibronectin-1, and IL-6 mRNA expression in LX2-HepG2 co-cultures in 3D healthy and cirrhotic liver scaffolds. Importantly, Sorafenib treatment reduced STAT3 phosphorylation in co-cultures engrafted both types of 3D scaffolds and abrogated TGF-β1-induced STAT3 phosphorylation.

An additional novel set of information provided by the present study is related to the validation of the 3D scaffold model as a new platform to test anti-cancer drugs and their mechanisms, and whether the 3D ECM scaffold would better mimic, or predict, processes such as cancer chemoresistance [14,55,56]. Indeed, previous studies reported that the 3D microenvironment of fibrotic liver promoted HCC cells progression and chemoresistance [57,58] and demonstrated that ECM-producing stromal cells are able to induce drug resistance and tumor progression in HCC cells within a 3D spheroid co-culture model [1,2,14,26]. Moreover, studies showed that HCC cells cultured in 3D tumor spheroids were able to enhance HCC characteristics and had greater resistance to various anti-cancer drugs than 2D cultures [14,55,56].

Along these lines, we opted to use Regorafenib, a novel oral multiple kinase inhibitor approved as a second-line drug for advanced HCC patients with tumor progression after Sorafenib treatment [3]. Regorafenib potentially inhibits tumor progression through suppression of angiogenesis, oncogenesis, and TME [59]. In this study, we demonstrated higher resistance to Regorafenib therapy (IC_50_, 16.6 µM for 6 days) when co-cultures were grown in 3D cultures versus 2D cultures (IC_50_, 3.6 µM for 2 days). In HCC, EMT plays a crucial role in tumor progression and metastasis [60] and TGF-β1 is a potent inducer of EMT through canonical (Smad2/3 dependent) and non-canonical pathways as we and others have described [12,61]. Previous studies have shown that Regorafenib inhibited TGF-β1-induced EMT in colorectal cancer by activating SHP-1-dependent P-STAT3 by directly dephosphorylating STAT3 on Tyr705 thus silencing the downstream pathway in in vivo and in vitro 2D models [62]. In HCC, Regorafenib is known to induce tumor cell apoptosis in 2D HCC cell culture by activating SHP-1-dependent P-STAT3 suppression thus SHP1 acts as a classical tumor suppressor [63]. However, recent studies have shown that levels of SHP-1 are significantly downregulated in human HCC tissues and reduced SHP-1 expression was associated with shorter overall survival of patients [64]. Our data demonstrated that Regorafenib induces apoptosis in co-cultures engrafting both 3D healthy and cirrhotic scaffolds. Moreover, we demonstrated that Regorafenib’s inhibitory effect on TGFβ1-induced EMT markers, occurred through suppression of P-STAT3, which coincided with the induction of E-cadherin protein expression, downregulation in vimentin expression, and a significant downregulation in SHP-1 in co-cultures grown in healthy and cirrhotic 3D scaffolds. Moreover, in this study, we demonstrated ECM-specific effects such as the significant upregulation of STAT3/P-STAT3 in non-treated control co-cultures engrafting 3D cirrhotic scaffolds in comparison to 3D healthy scaffolds. Nevertheless, Regorafenib abolished the phosphorylation of STAT3 in both types of 3D scaffolds. SHP-1 expression showed ECM-dependent effects as SHP-1 protein expression was significantly downregulated in non-treated control co-cultures in 3D cirrhotic scaffolds versus 3D healthy scaffolds, confirming a possible negative effect of the ECM microenvironment on cell behavior as SHP-1 expression levels are significantly downregulated in human HCC tissues [64]. Thus, Regorafenib could abolish SHP-1 protein expression in co-cultures engrafted in 3D healthy scaffolds but not 3D cirrhotic scaffolds.

## 5. Conclusions

The results of the study suggest that this newly proposed 3D co-culture platform is able to mimic the natural physio-pathological microenvironment and could be employed for anti-fibrotic and anti-HCC drug screening.

## Data Availability

The datasets used and analyzed during the current study are available from the corresponding author on reasonable request.

## Supplementary Materials

The following are available online at https://www.mdpi.com/article/10.3390/cancers13194936/s1, Table S1: Decellularization protocol, Table S2: Primary antibodies for immunohistochemistry staining, Table S3: Applied Biosystems Taqman Gene Expression Assay, Table S4: Primary and secondary antibody for western blot analysis, Table S5: The densitometry readings/intensity ratio of studied western blot, Figure S1: Whole blot showing all the bands with all molecular weight markers on the Western, Figure S2: H&E staining of co-cultures repopulating the healthy and cirrhotic 3D scaffolds exposed to Regorafenib and TGF-β1.
