# Supplementary material for: Optimization and Validation of a Novel Three-Dimensional Co-Culture System in Decellularized Human Liver Scaffold for the Study of Liver Fibrosis and Cancer
Source: Cancers (Basel). 2021 Sep 30;13(19):4936. doi: 10.3390/cancers13194936 (PMC8508071; doi:10.3390/cancers13194936)

Supplementary Figure **Whole blot showing all the bands with all molecular weight markers on the Western**

<u>Figure 2A</u>

PDGF-B

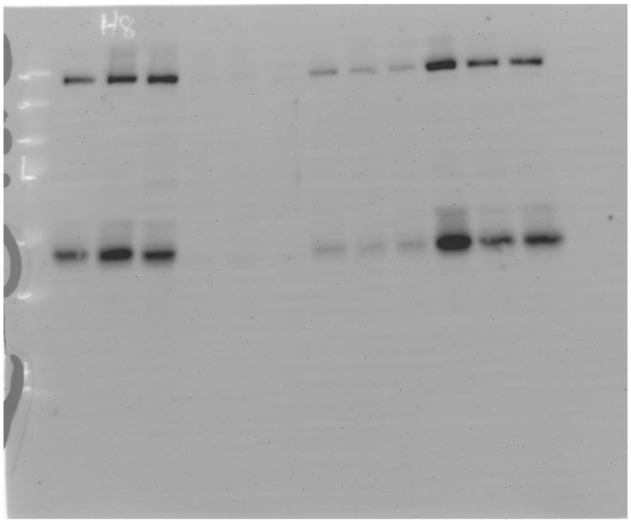

GAPDH

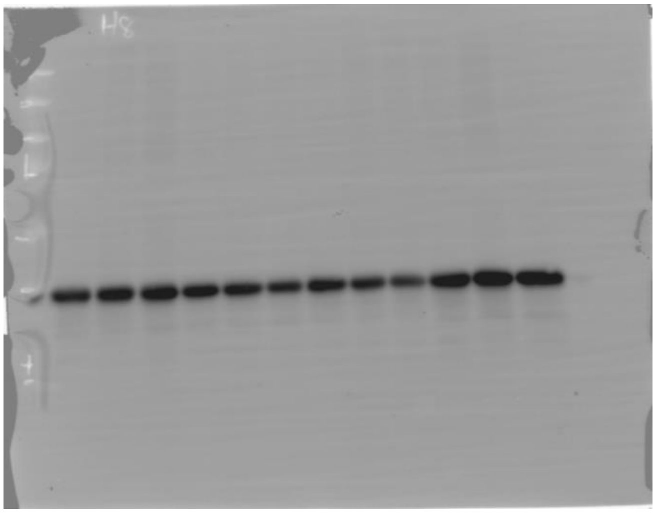

EPCAM

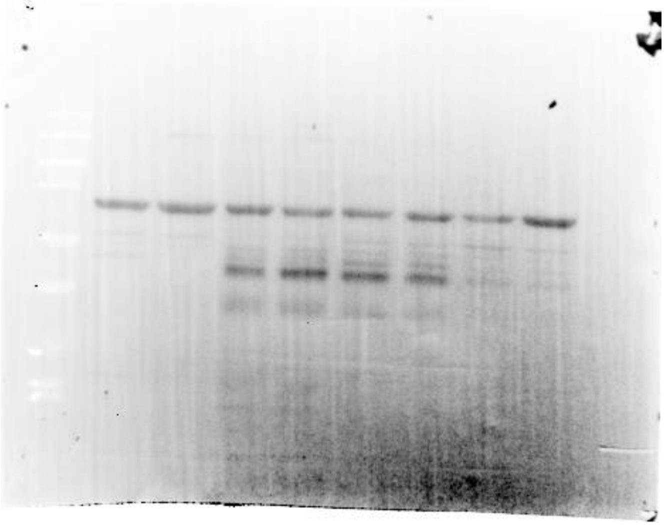

GAPDH

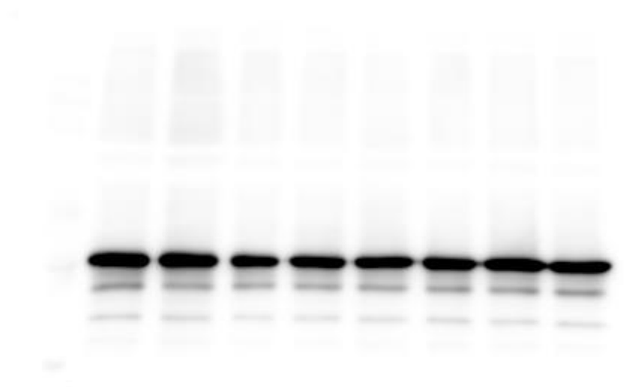

Figure 4D (Healthy scaffold)

PDGF-B

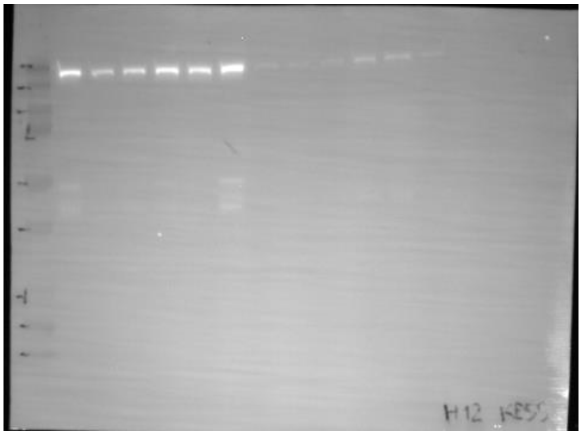

STAT3

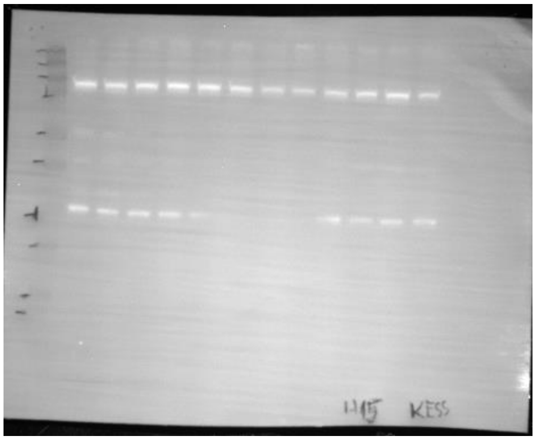

GAPDH

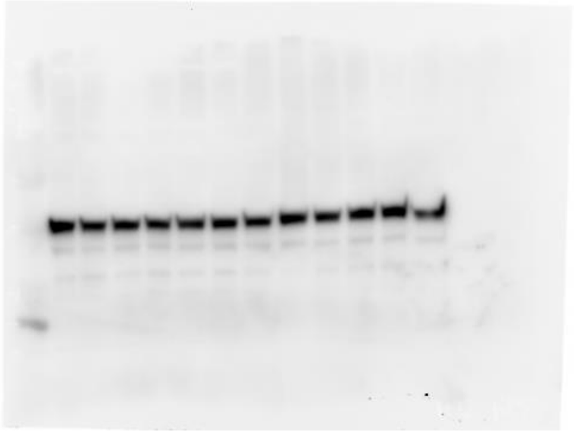

GAPDH

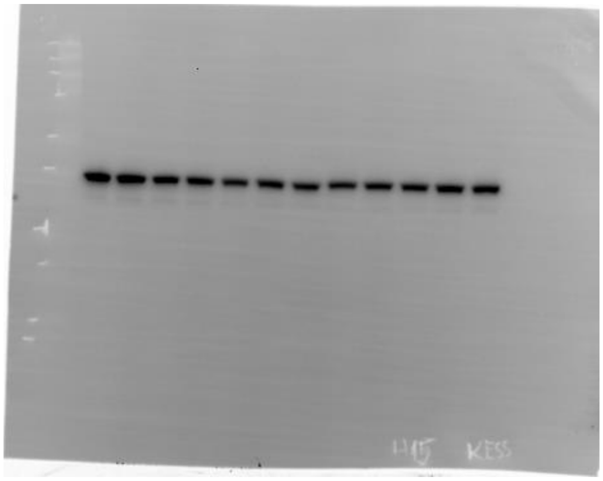

PSTAT3

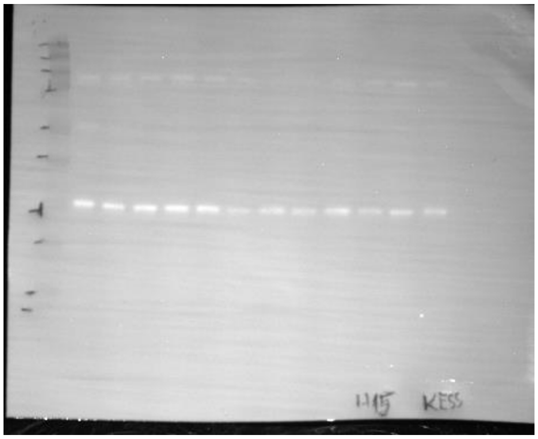

__Figure 4D (Cirrhotic scaffold)__

PDGF-B

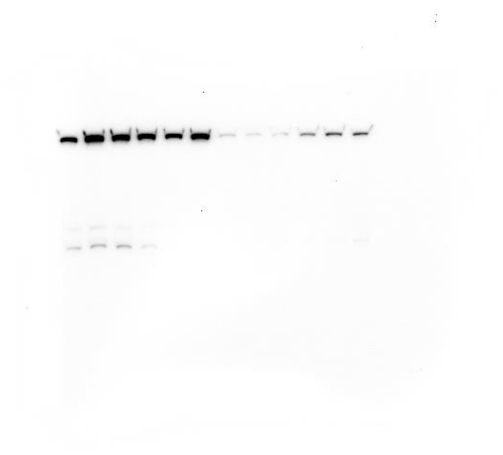

GAPDH

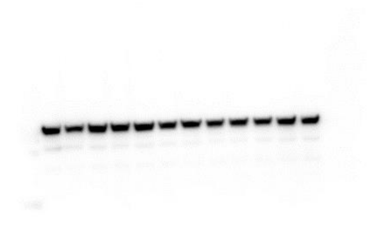

PSTAT3

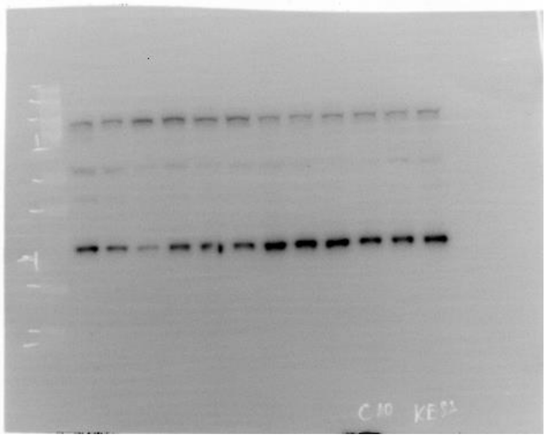

STAT3

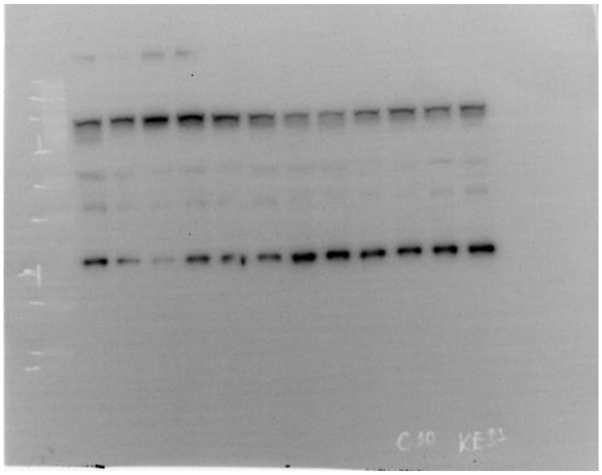

GAPDH

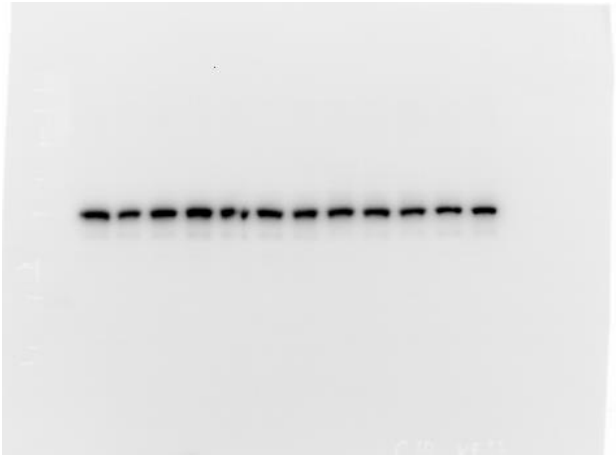

Figure 5B

Cleave PARP-1

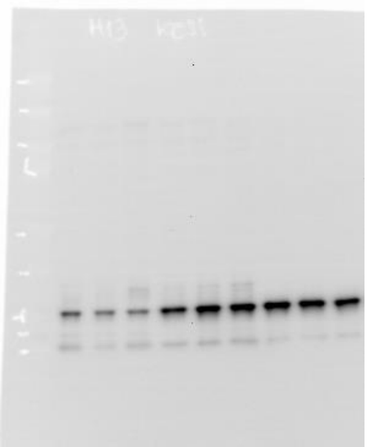

a-Tubulin

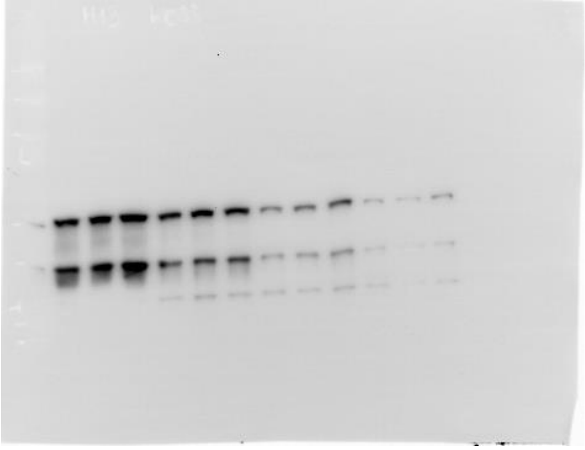

PSTAT3

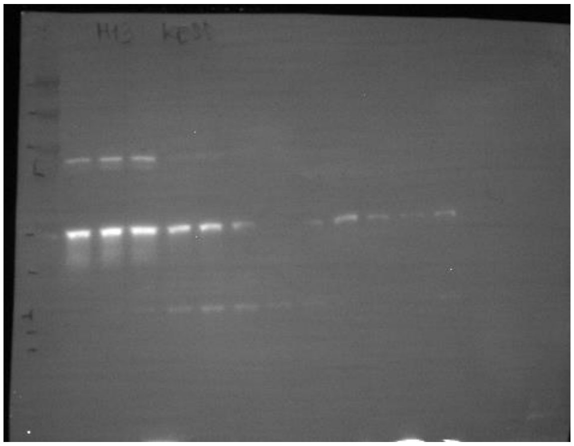

GAPDH

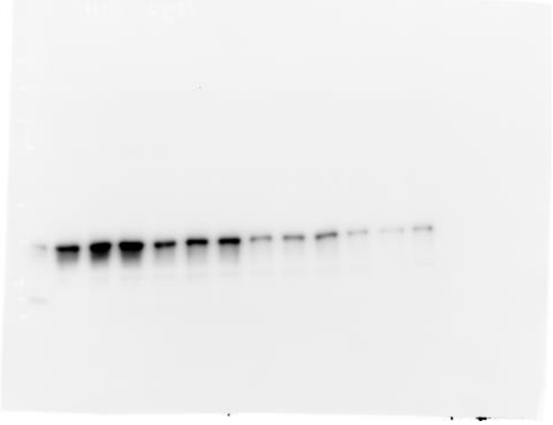

STAT3

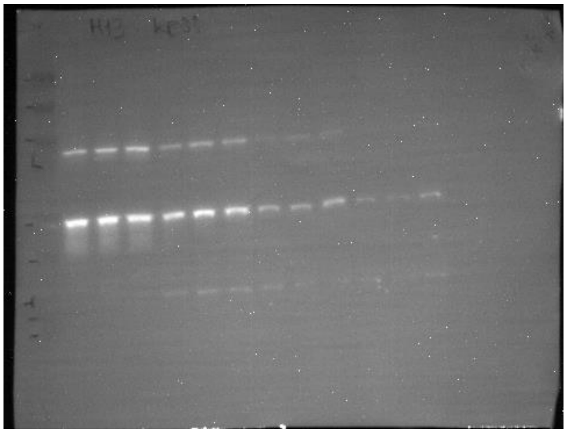

Histone H3

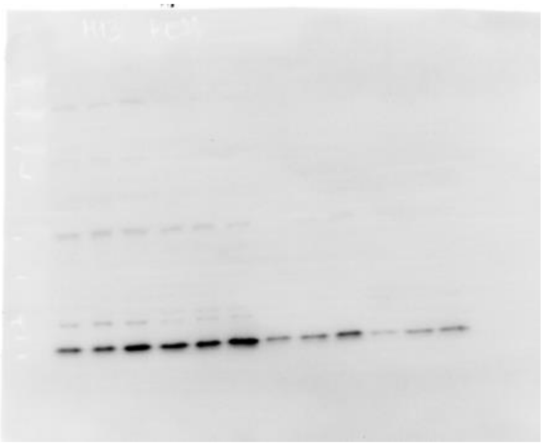

Figure 6A

SHP-1

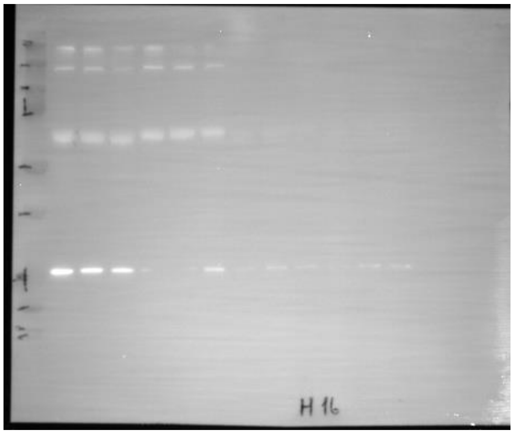

E-cadherin

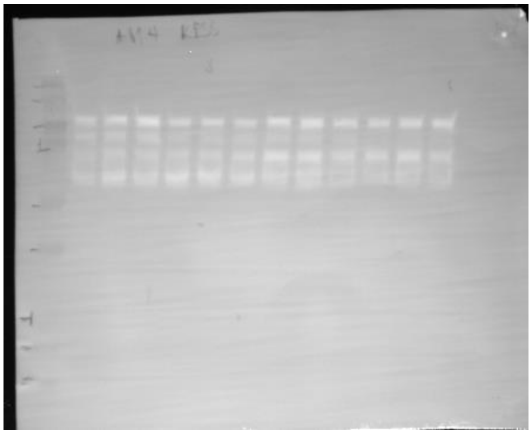

PSTAT3

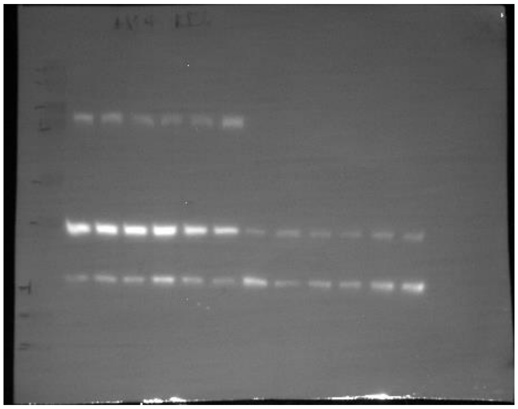

Vimentin

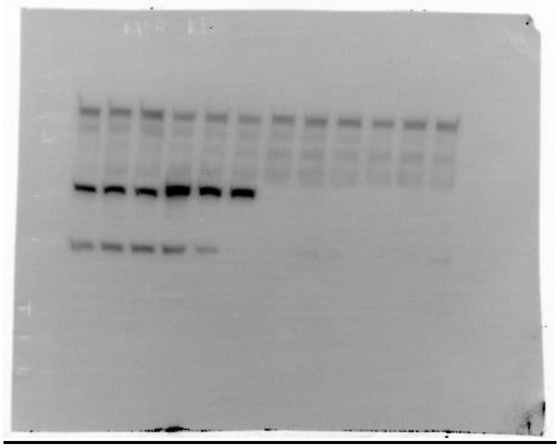

STAT3

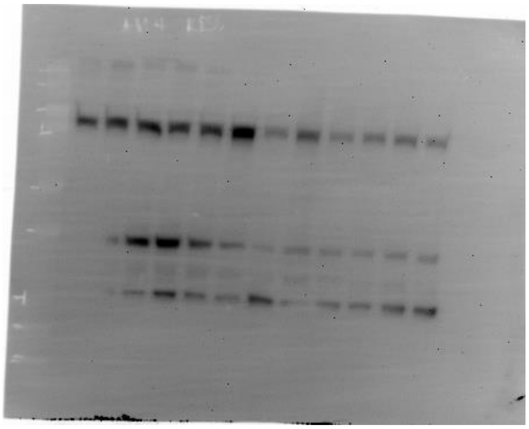

Figure 6A (continue)

GAPDH

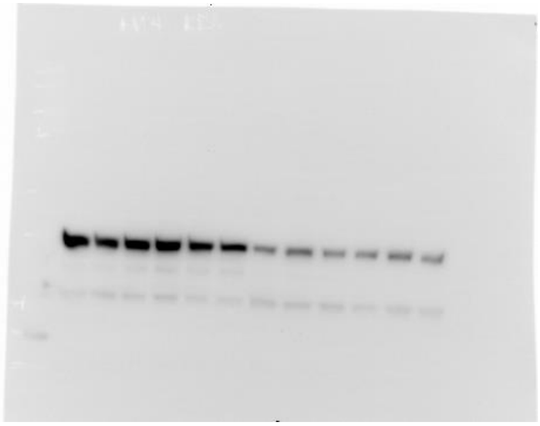

Histone H3

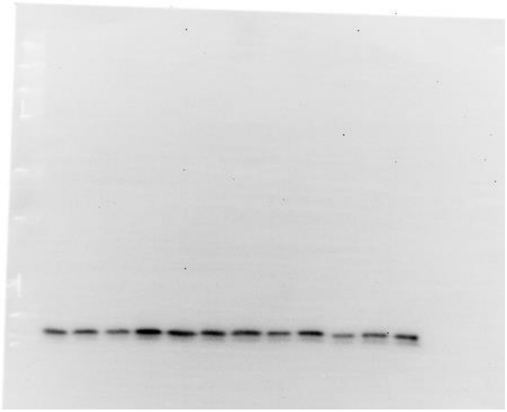

Figure 6B

SHP-1

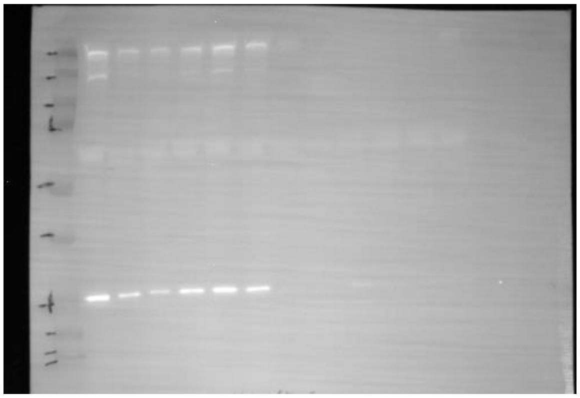

E-cadherin

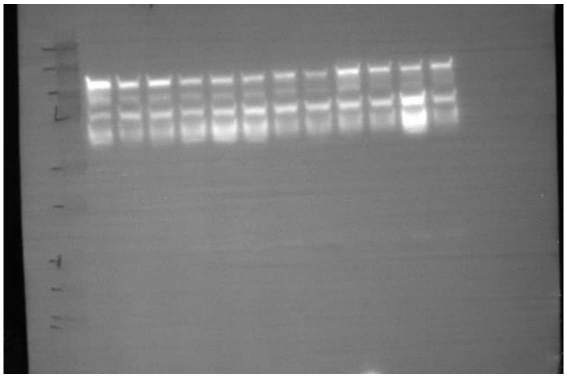

PSTAT3

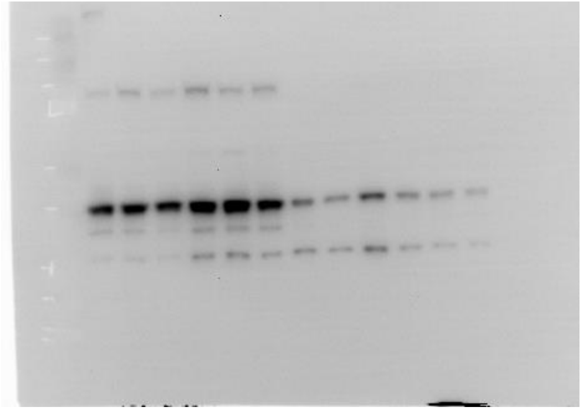

Vimentin

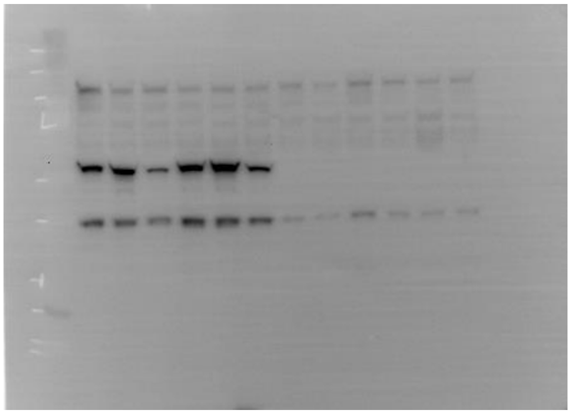

STAT3

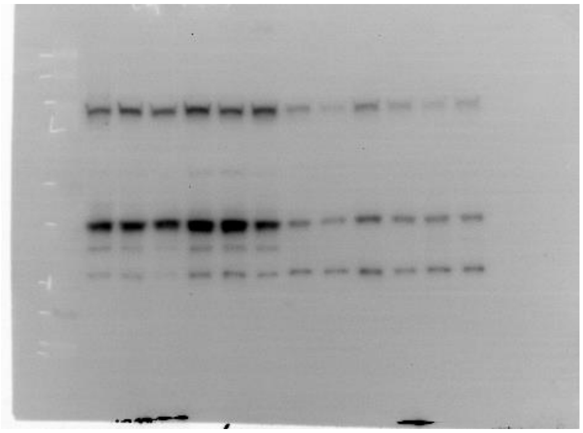

Figure 6B (continue)

GAPDH

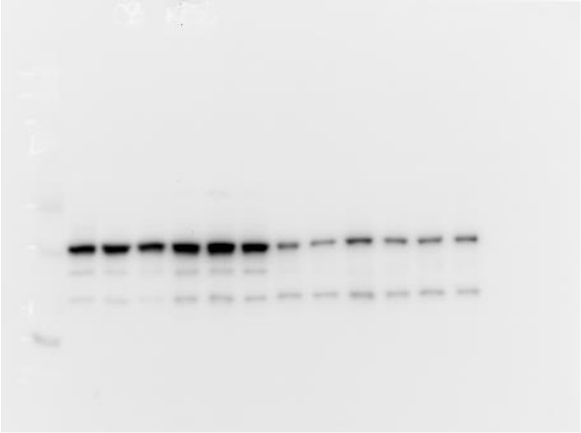

Histone H3

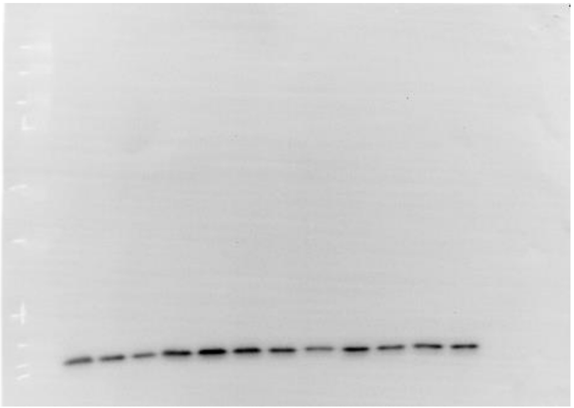

Figure 7

SHP-1

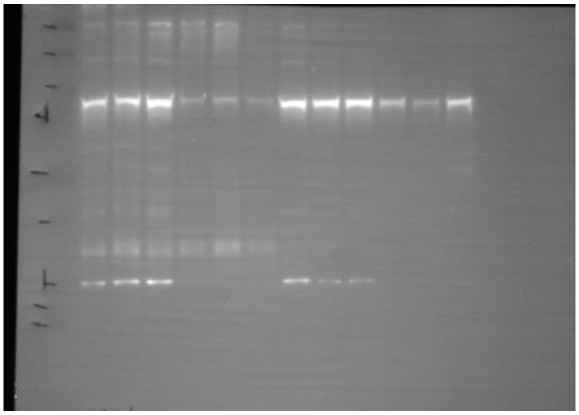

PSTAT3

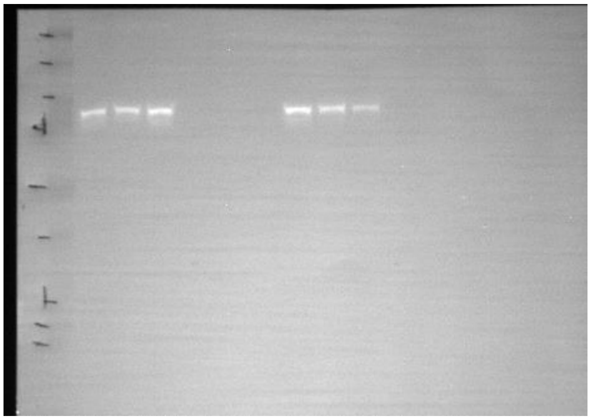

STAT3

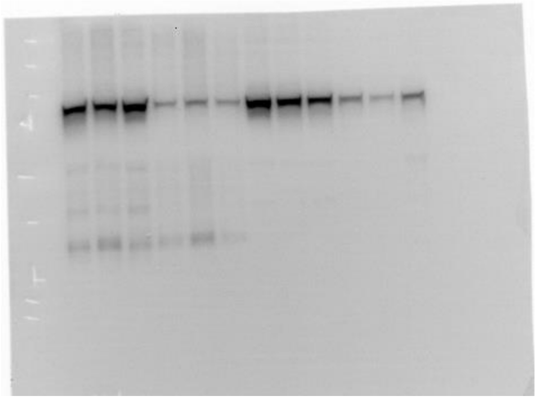

GAPDH

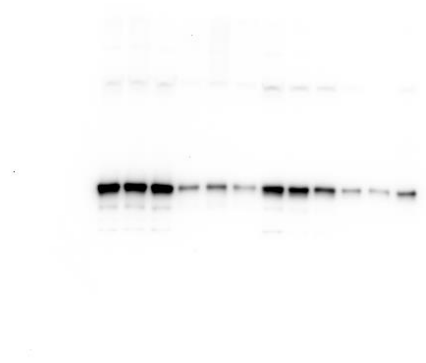

Histone H3

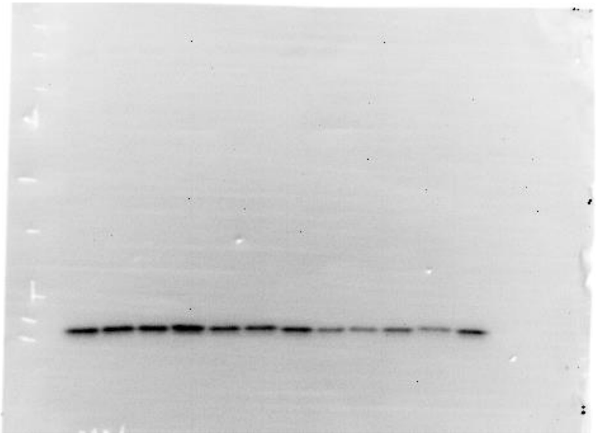

Supplement: Supplementary file 1 [file cancers-13-04936-s001.zip › Supplementary Files/Supplementary Figure S1.pdf]
