# Supplementary material for: Optimization and Validation of a Novel Three-Dimensional Co-Culture System in Decellularized Human Liver Scaffold for the Study of Liver Fibrosis and Cancer
Source: Cancers (Basel). 2021 Sep 30;13(19):4936. doi: 10.3390/cancers13194936 (PMC8508071; doi:10.3390/cancers13194936)

Supplementary table 1: Decellularization protocol

Type of scaffold	Step	Solution	Frequency of oscillation (Hertz)	Time (minutes)	Repetitions
**Healthy scaffold**	1	Deionized water	20	2	12-18
	2	Phosphate buffered saline 1X	20	2	1-2
**Cirrhotic scaffold**	1	Deionized water	30	5	7
	2	Reagent mixture	30	5	2
	3	Deionized water	30	5	4
	4	Reagent mixture	30	5	2
	5	Deionized water	30	5	4
	6	Reagent mixture	30	5	2
	7	Deionized water	30	5	4
	8	Reagent mixture	30	5	2
	9	Deionized water	30	5	4
	10	Reagent mixture	30	5	2
	11	Deionized water	30	5	4
	12	Reagent mixture	30	5	2
	13	Deionized water	30	5	4
	14	Reagent mixture	30	5	2
	15	Phosphate buffered saline 1X	30	5	5

Supplementary table 2: Primary antibodies for immunohistochemistry staining

**Antibody**	**Species**	**Company**	**Catalogue number**	**Dilution**
Platelet derived growth factor receptor-beta (PDGFR-β)	Rabbit	Abcam	Ab32570	1: 75
Epithelial Cell Adhesion Molecule (EPCAM)	Rabbit	Abcam	Ab71916	1:50
Ki-67	Rabbit	Abcam	Ab21700	Pre-diluted

Supplementary table 3: Applied Biosystems Taqman Gene Expression Assay.

Gene Name	Gene Abbreviation	Taqman Gene Assay No.
Albumin	ALB	Hs00609411_m1
Alpha-fetoprotein	AFP	Hs01040598_m1
Collagen Type 1, alpha 1 chain	Col1A1	Hs00164004_m1
Collagen Type 3, alpha 1 chain	Col3A1	Hs00943809_m1
Cytochrome P450 3A4	CYP3A4	Hs00604506_m1
Fibronectin-1	FN-1	Hs01549976_m1
Glyceraldehyde-3-phosphate dehydrogenase	GAPDH	Hs02758991_m1
Hepatocyte nuclear factor 4 alpha	HNF4a	Hs00230853_m1
Interleukin-6	IL-6	Hs00985639_m1
Lysyl oxidase	LOX	Hs00942480_m1
Platelet-derived growth factor receptor beta	PDGFR-β	Hs01019589_m1
Transforming growth factor, beta 1	TGFβ1	Hs00998133_m1
Uridine 5'-diphospho glucuronosyltransferase family 1 member A1	UGT1A1	Hs02511055_m1

Supplementary table 4: Primary and secondary antibody for western blot analysis

Type	Antibody	Species	Company	Dilution
**Primary antibody**	Alpha-tubulin	Rabbit	Cell Signaling	1:1,000
	Cleaved PARP-1	Rabbit	Abcam	1:2,500
	E-cadherin	Rabbit	Cell Signaling	1:1,000
	EPCAM	Rabbit	Abcam	1:1,000
	GAPDH	Mouse	Santa Cruz Biotechnology	1:200
	PDGFR-β	Rabbit	Abcam	1:5,000
	P-STAT3	Rabbit	Cell Signaling	1:1,000
	SHP-1	Rabbit	Abcam	1:1,000
	STAT3	Rabbit	Cell Signaling	1:1,000
	Vimentin	Mouse	Abcam	1:1,000
	Histone	Rabbit	Cell Signaling	1:1,000
**Secondary antibody**	Anti-rabbit IgG HRP	Goat	Santa Cruz Biotechnology	1:10,000
	Anti-mouse IgG HRP	Goat	Santa Cruz Biotechnology	1:10,000

Supplementary table 5: The densitometry readings/intensity ratio of studied western blot

Figure 2A	Order	Net PDGF-B/GAPDH	Net EPCAM/GAPDH
**LX2**	**1**	1.530471479	0.276075
	**2**	0.935325263	0.234582
	**3**	0.691017018	**-**
**HEPG2**	**1**	0.086857978	0.652503
	**2**	0.080116802	0.713105
	**3**	0.064155385	**-**
**SimCC**	**1**	0.434645048	0.766328
	**2**	0.172312701	0.785488
	**3**	0.26151477	**-**
**SeqCC**	**1**	0.868664775	0.574125
	**2**	0.979548709	0.371595
	**3**	0.688862919	**-**
**Healthy scaffold (Figure 4D)**	**Order**	**Net PDGF-B/GAPDH**	**Net PSTAT3/GAPDH**
**Control**	**1**	0.546	0.448534
	**2**	0.569774	0.325819
	**3**	0.545869	0.506053
**TGF-β1**	**1**	0.722312	0.570713
	**2**	0.628336	0.587459

	**3**	0.514984	0.387194
**Sorafenib**	**1**	0.174727	0.15549
	**2**	0.064897	0.156268
	**3**	0.078708	0.22321
**TGF-β1 + Sorafenib**	**1**	0.197797	0.294274
	**2**	0.130617	0.350418
	**3**	0.119314	0.155967
**Cirrhotic scaffold (Figure 4D)**	**Order**	**Net PDGF-B/GAPDH**	**Net PSTAT3/GAPDH**
**Control**	**1**	1.575095	0.306686
	**2**	1.429771	0.349057
	**3**	1.329299	0.405718
**TGF-β1**	**1**	1.328629	0.410499
	**2**	1.23693	0.471446
	**3**	0.957926	0.456607
**Sorafenib**	**1**	0.335779	0.264145
	**2**	0.302115	0.200761
	**3**	0.300607	0.218723
**TGF-β1 + Sorafenib**	**1**	0.439778	0.256304
	**2**	0.831272	0.206496
	**3**	0.693803	0.319257
**Figure 5B**	**Order**	**Net cleave PARP-1/Histone H3**	**Net PSTAT3/HistoneH3**
**Control**	**1**	1.163119	0.869111
	**2**	0.888266	0.992323
	**3**	0.525068	0.57134
**10 µM**	**1**	1.631586	0.103875
	**2**	1.963694	0.113145
	**3**	1.52087	0.058874
**20 µM**	**1**	6.683193	0.02194
	**2**	5.79775	0.01694
	**3**	2.998871	0.01144

**Healthy scaffold (Figure 6A)**	**Order**	**Net PSTAT3/Histone H3**	**Net SHP-1/ Histone H3**	**Net E-cadherin/ Histone H3**	**Net Vimentin/Histone H3**
**Control**	**1**	0.774449	1.91216	1.711508	1.385469
	**2**	0.637865	1.832319	1.961001	1.266615
	**3**	0.511088	2.242285	3.020526	1.324894
**TGF-β1**	**1**	0.265599	1.336909	0.711498	1.70726
	**2**	0.264304	1.536941	0.795431	1.649943

	**3**	0.617372	1.140336	0.634874	1.877293
**Regorafenib**	**1**	0.00852	0.194104	1.579572	0.016445
	**2**	0.043181	0.349874	2.485745	0.012599
	**3**	0.00024	0.16396	1.507882	0.015329
**TGF-β1 + Regorafenib**	**1**	0.02289	0.293735	2.177803	0.034472
	**2**	0.010019	0.247217	1.987521	0.023993
	**3**	0.00112	0.223675	1.772502	0.024936
**Cirrhotic Scaffold (Figure 6B)**	**Order**	**Net PSTAT3/Histone H3**	**Net SHP-1/ Histone H3**	**Net E-cadherin/ Histone H3**	**Net Vimentin/Histone H3**
**Control**	**1**	0.13178	1.224588	2.630614	1.99736
	**2**	0.334	0.657593	1.440182	2.013163
	**3**	0.234048	0.893641	2.317789	1.201311
**TGF-β1**	**1**	0.416526	0.545129	0.519528	0.0123
	**2**	0.201932	0.483679	0.531781	0.02169
	**3**	0.290255	0.424768	0.527973	0.00978
**Regorafenib**	**1**	0.00769	0.356404	0.958175	0.0123
	**2**	0.001189	0.38885	0.784613	0.02169
	**3**	0.00348	0.29649	1.543068	0.00978
**TGF-β1 + Regorafenib**	**1**	0.010488	0.464304	1.495245	0.01247
	**2**	0.010082	0.534939	1.055324	0.01145
	**3**	0.00193	0.461609	1.230926	0.00451

**Figure 7**	**Order**	**Net PSTAT3/Histone H3**	**Net SHP-1/ Histone H3**
**Control healthy scaffold**	**1**	1.592528	0.294782
	**2**	1.501621	0.288089
	**3**	1.812561	0.266128
**Regorafenib healthy scaffold**	**1**	0.015527	-0.0094
	**2**	0.013088	0.053523
	**3**	0.018406	0.005491
**Control cirrhotic scaffold**	**1**	2.199337	0.110006
	**2**	3.712782	0.02515
	**3**	3.326216	0.016434
**Regorafenib cirrhotic scaffold**	**1**	0.023959	-0.03678
	**2**	-0.0178	-0.01881
	**3**	0.012295	0.235535

Supplementary figure [illegible] H&E staining of co-cultures repopulating the healthy and cirrhotic 3D scaffolds exposed to Regorafenib and TGF-β1

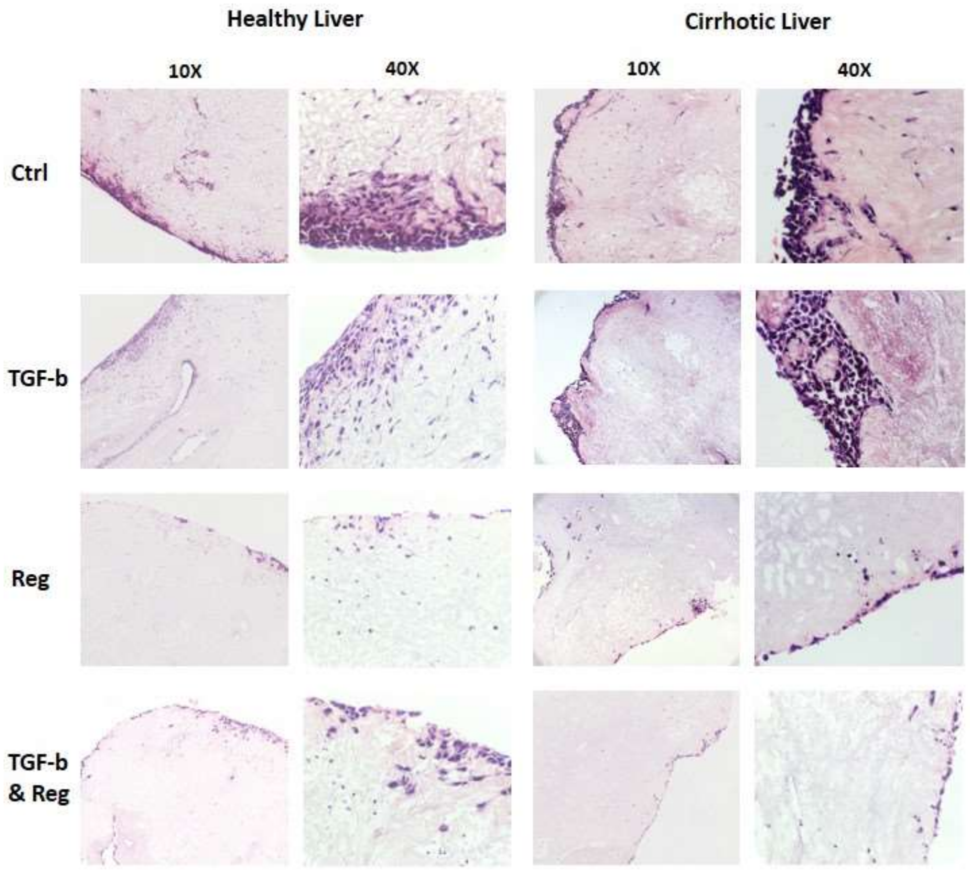

Supplement: Supplementary file 1 [file cancers-13-04936-s001.zip › Supplementary Files/Supplementary Tables and Figure S2.pdf]
